# What is living on your dog's skin? Characterization of the canine cutaneous mycobiota and fungal dysbiosis in canine allergic dermatitis

**DOI:** 10.1093/femsec/fiv139

**Published:** 2015-11-05

**Authors:** Courtney Meason-Smith, Alison Diesel, Adam P. Patterson, Caitlin E. Older, Joanne M. Mansell, Jan S. Suchodolski, Aline Rodrigues Hoffmann

**Affiliations:** 1 Dermatopathology Specialty Service, Department of Veterinary Pathobiology, College of Veterinary Medicine and Biomedical Sciences, Texas A&M University, College Station, TX 77843-4467, USA; 2 Department of Small Animal Clinical Sciences, College of Veterinary Medicine and Biomedical Sciences, Texas A&M University, College Station, TX 77843-4474, USA

**Keywords:** fungi, microbiome, dog, skin, ITS, atopic dermatitis

## Abstract

To characterize the skin-associated fungal microbiota (mycobiota) in dogs, and to evaluate the influence of body site, individual dog or health status on the distribution of fungi, next-generation sequencing was performed targeting the internal transcribed spacer region. A total of 10 dogs with no history of skin disease were sampled at 10 distinct body sites consisting of haired and mucosal skin, and 8 dogs with diagnosed skin allergies were sampled at six body sites commonly affected by allergic disease. Analysis of similarities revealed that body site was not an influencing factor on membership or structure of fungal communities in healthy skin; however, the mucosal sites were significantly reduced in fungal richness. The mycobiota from body sites in healthy dogs tended to be similar within a dog, which was visualized in principle coordinates analysis (PCoA) by clustering of all sites from one dog separate from other dogs. The mycobiota of allergic skin was significantly less rich than that of healthy skin, and all sites sampled clustered by health status in PCoA. Interestingly, the most abundant fungi present on canine skin, across all body sites and health statuses, were *Alternaria* and *Cladosporium*—two of the most common fungal allergens in human environmental allergies.

## Abstract (precis)

The skin-associated fungal microbiota were characterized in healthy dogs and those with skin allergies using next-generation sequencing, and the authors identified significant influences of the dog and health status on the distribution and diversity of fungal communities.

## Graphical abstract

**Graphical Abstract Figure.**
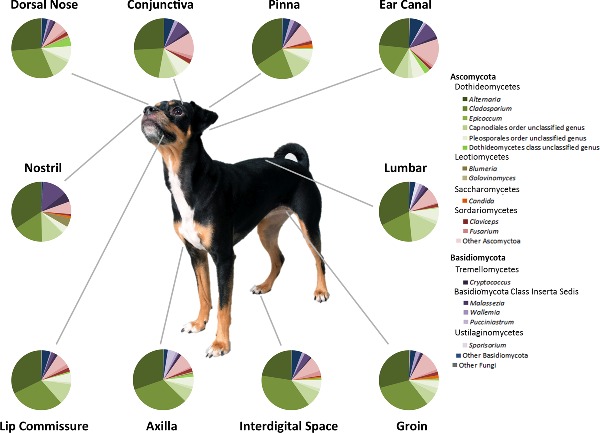
The skin-associated fungal microbiota were characterized in healthy dogs and those with skin allergies using next-generation sequencing, and the authors identified significant influences of the dog and health status on the distribution and diversity of fungal communities.

## INTRODUCTION

Skin diseases are often characterized by multifaceted etiology with potential contributions coming from the host's genetics, skin barrier integrity, immune system and inflammatory components, which can be exacerbated by environmental exposure and hygiene practices (Human Microbiome Project 2012). The cutaneous microbiota associated with skin diseases have only recently been investigated in humans. Through this work, dysbiosis (an alteration to the normal microbiota) of cutaneous microbiota has been associated with a variety of human skin diseases including psoriasis (PS) (Alekseyenko *et al*. 2013; Takemoto *et al*. 2015), acne vulgaris (Fitz-Gibbon *et al*. 2013) and atopic dermatitis (AD) (Kong *et al*. 2012; Oh *et al*. 2013). Fewer studies have focused on how the microbiota influences skin health of other host species such as dogs (Rodrigues Hoffmann *et al*. 2014). In addition to improving animal health, these studies are needed to evaluate how animal behavior, anatomy or environmental exposure affects cutaneous microbiota, and ultimately health status of the host. Interest in the microbial communities of companion animals is growing as we begin to recognize how their microbiota can influence our own (Song *et al*. 2013) and possibly affect human health for people cohabiting with pets (Misic *et al*. 2015).

AD is a pruritic condition characterized by a skin barrier dysfunction and hypersensitization to environmental allergens (Kondo, Ichikawa and Imokawa 1998; Miller *et al*. 2013b). In both people and dogs, there are increased levels of IgE to environmental allergens, an initial type I hypersensitivity reaction characterized by increased numbers of T-helper type-2 (Th2) cells in lesional skin and later type IV hypersensitivity in chronic cases, supporting a similar pathogenesis of AD in these two host species (Miller *et al*. 2013b). Fungi are generally thought to be less influential in the pathogenesis of AD; however, *Malassezia* hypersensitivity has been implicated in both human and canine AD through patch testing, IgE studies and responsiveness to antifungal therapy (Morris, Olivier and Rosser 1998; Farver *et al*. 2005; Bond *et al*. 2006; Casagrande *et al*. 2006; Kato *et al*. 2006; Zhang *et al*. 2011a). Recently, next generation sequencing (NGS) studies have identified bacterial dysbiosis associated with affected human skin in AD (Kong *et al*. 2012) and PS (Alekseyenko *et al*. 2013), and with non-affected skin in dogs with allergic skin disease, which includes AD (Rodrigues Hoffmann *et al*. 2014). Bacterial dysbiosis associated with AD and PS is characterized by a reduction of bacterial diversity in affected skin, and shifts in relative abundance of particular bacterial species (Kong *et al*. 2012; Alekseyenko *et al*. 2013). Fungal dysbiosis has also been reported for these diseases in humans, but instead of reduced diversity as found for the bacterial microbiota, there is an increase in fungal diversity at the site of lesions (Zhang *et al*. 2011b; Oh *et al*. 2013; Takemoto *et al*. 2015), and clustering by health status in principle coordinates analysis (PCoA) (Zhang *et al*. 2011b; Oh *et al*. 2013; Takemoto *et al*. 2015).

Prior to investigating how a disease process has altered the host microbiota, or if the microbiota might play a role in disease pathogenesis, there must be the initial studies of healthy skin microbiota and determination of the factors influencing their ecological distribution and function. A few studies have characterized the fungal microbiota (mycobiota) of human skin using NGS (Zhang *et al*. 2011b; Park *et al*. 2012; Findley *et al*. 2013; Oh *et al*. 2013; Jung *et al*. 2015; Takemoto *et al*. 2015). One study revealed that healthy human skin is predominantly colonized by the genus *Malassezia* with body site differences seen only for the different species of *Malassezia* (Findley *et al*. 2013). Fungal diversity was dependent upon body site, and the greatest diversity was found in samples from feet. Retesting of individuals over time demonstrated a stable fungal community structure at the core and arm body sites, but not at the feet. Overall fungal community structure was strongly correlated with the site location (head, torso, arms, feet) (Findley *et al*. 2013), in contrast to bacterial communities that are more dependent on site physiology (dry, moist, sebaceous) (Grice *et al*. 2009).

We are still in the early stages of describing the skin microbiota in companion animals using NGS. To date there has been one NGS study published by our group characterizing the bacterial microbiota of canine skin (Rodrigues Hoffmann *et al*. 2014). Similar to human skin, bacterial community composition is significantly different between body sites. The predominant phylum across all body sites in dogs is *Proteobacteria*, unlike human skin that is predominantly colonized by *Actinobacteria* and *Firmicutes* (Grice *et al*. 2009). Richness and diversity of bacterial taxa varies across the canine body sites with the nostril and conjunctiva harboring the fewest, and the dorsal nose the greatest (Rodrigues Hoffmann *et al*. 2014).

Descriptions of the mycobiota in dogs using NGS have been limited to the fecal mycobiota in healthy dogs and those with diarrhea (Foster *et al*. 2013). The only studies aimed at characterizing fungi on the skin of dogs have been culture based (Kennis *et al*. 1996; Yoshida, Naito and Fukata 2002; Dizotti and Coutinho 2007; Lyskova, Vydrzalova and Mazurova 2007; Oliveira *et al*. 2008; Campbell *et al*. 2010; Verneuil *et al*. 2014). One study sampled strictly the ears of dogs (*n* = 194) that were either healthy, atopic or had otitis (Campbell *et al*. 2010). In this study, the most abundant fungal organism cultured was *Penicillium* spp., and the second was *Malassezia pachydermatis*. Another study from France cultured fungi from the conjunctiva and adjacent skin on the nose of dogs and identified the presence of additional fungi including *Alternaria*, *Cladosporium* and *Aspergillus* (Verneuil *et al*. 2014). While these studies are all valuable, it is well documented that molecular-based studies provide a more comprehensive picture of the microbial landscape due to the non-cultivable nature of some microbes or “selective” culture of others (Stewart 2012).

The goal of this study was to characterize the canine cutaneous mycobiota using NGS, and determine whether body sites influenced the distribution of fungal organisms. A total of 10 distinct body sites, consisting of haired skin, mucosal surfaces and one mucocutaneous junction were sampled in 10 healthy dogs. We expected to find a greater diversity of fungal commensals than what has been detected by fungal culture alone. Similar to human skin, we expected to see a dependence of fungal communities on site location. To additionally investigate the role of the mycobiota in canine allergic skin disease, we also collected skin swabs from eight dogs with allergic skin disease at six body sites that are commonly affected by cutaneous manifestation of allergies. We expected to find changes in the diversity, membership and structure of fungal communities, as well as increased abundances of *M. pachydermatis* owing to its implication in canine AD.

## MATERIALS AND METHODS

### Subject recruitment

All samples for this study were collected following a protocol approved by the Texas A&M University Institutional Animal Care and Use Committee. A total of 10 dogs (D1-D10) with no history of skin disease were recruited for collection of healthy skin samples (Table 1). These dogs ranged from 1.5 to 11 years old, and included five castrated males and five spayed females. There were four mixed breed dogs, two Jack Russell Terriers, one Beagle, one Pitbull, one Boston Terrier and one German Shepherd. A board certified veterinary dermatologist clinically evaluated 10 healthy dogs, and also evaluated 8 additional dogs (D11–D18) for inclusion in the allergic group (Table 1). Six dogs were diagnosed with AD using standard diagnostic methods including fulfillment of Favrot's criteria and exclusion of other pruritic dermatoses (Olivry *et al*. 2010). One dog was diagnosed with chronic pododermatitis and cutaneous adverse food reactions (CAFR), and one dog was diagnosed with only CAFR. The allergic dogs ranged from 2 to 10 years old, included four castrated males, and four spayed females. The breeds of allergic dogs were two Boston Terriers, two Cavalier King Charles Spaniels, one Shetland Sheepdog, one Australian Shepherd, one Labrador retriever and one mixed breed dog. To be included in the study, dogs could not have displayed overt clinical signs of bacterial or fungal skin infections at the time of sample collection. Five out of eight allergic dogs were receiving medication for their AD: oral immunotherapy (2), oclacitinib (Apoquel^®^, Zoetis) (2) and oral cyclosporine (Atopica, Novartis) (1). All healthy study participants did not receive systemic antibiotics or antifungals 6 months prior to collection of samples, and allergic study participants 1 month prior. Additionally, no dog was allowed to be bathed 1 week prior to the beginning of the study. Healthy dogs had not received steroids previously, and all but one of the allergic dogs had not received steroids within the last month prior to the study. Allergic dogs were allowed to have their allergic disease managed with either long-term medication and/or immunotherapy without the need for withdrawal for study inclusion purposes.

**Table 1.** Medical histories and environmental exposures of dogs enrolled in this study. Allergy pruritus, ear problems and fleas were part of the clinical history and not clinically present at the time of sample collection.

Dog	Health status	Breed	Age	Sex	Allergy pruritis	Ear problems	Fleas	Time indoors	Outdoor environment	Indoor environment	Allergy treatments	Steroids	Previous antibiotic usage
D1	Healthy	Jac	9	M	N	N	N	80	TGW	CTFB	N/A	N	Y
D2	Healthy	Mix	1.5	M	N	N	N	90	GW	CTFB	N/A	N	N
D3	Healthy	Mix	2	M	N	N	N	80	TGW	CTFB	N/A	N	N
D4	Healthy	Mix	3.5	M	N	N	N	90	TGW	CTFB	N/A	N	N
D5	Healthy	Bea	2	M	N	N	N	90	GW	CTFB	N/A	N	N
D6	Healthy	Mix	1.5	F	N	N	N	70	TGW	CTFB	N/A	N	N
D7	Healthy	Pit	9	F	N	N	Y	90	TGW	TF	N/A	N	N
D8	Healthy	Bos	3	F	N	N	Y	70	TGW	TF	N/A	N	N
D9	Healthy	Jac	11	F	N	N	Y	90	TGW	CTFB	N/A	N	N
D10	Healthy	Ger	7	F	N	N	N	50	TGW	CTFB	N/A	N	N
D11	Allergic	Bos	2	M	Y	Y	N	98	W	CTFB	Oral immunotherapy	Prednisone	N
D12	Allergic	Bos	7	M	Y	Y	N	98	W	CTFB	Oral immunotherapy	Prednisone	N
D13	Allergic	Mix	6	M	Y	Y	N	70	TGW	TFB	Oclacitinib	Prednisone	Y
D14	Allergic	Cav	2	F	Y	N	Y	99	TGW	CTFB		Y	Y
D15	Allergic	She	5	F	N	Y	N	95	TGW	CTFB	Cyclosporine	N	N
D16	Allergic	Aus	3	M	N	N	N	50	TGW	CTFB		Prednisone	N
D17	Allergic	Lab	10	F	Y	N	Y	95	TGW	T	Fluconazole	Prednisone	N
D18	Allergic	Cav	10	F	Y	Y	Y	90	TGW	TF	Oclacitinib	N	N

Jac: Jack Russell Terrier, Mix: Mixed breed, Bea: Beagle, Pit: Pitbull Terrier, Bos: Boston Terrier, Ger: German Shephard, Cav: Cavalier King Charles Spaniel, She: Shetland Sheepdog, Aus: Australian Shepherd, Lab: Labrador, T: Trees, G: Grass, W: Weeds, C: Carpet, T: Tile Floors, F: Furniture, B: Bedding, M: Male, F: Female, N: No, Y: Yes, and N/A: not available. Allergy treatments were concurrent, and D16 was the only dog currently taking steroids, all others with a Y had not received steroids in the last month.

### Sample collection and DNA extraction

A total of 10 body sites on healthy dogs were swabbed including the axilla, conjunctiva, dorsal nose, ear canal, groin, interdigital space, lip commissure, lumbar, nostril and pinna. Six body sites on allergic dogs that are commonly affected by cutaneous manifestation of allergies were swabbed including the axilla, ear canal, groin, interdigital space, lumbar and nostril. Samples were only collected from the right side of all dogs. Gloves were changed between dogs and the exam table was wiped down with DNA away (Molecular BioProducts, Inc., San Diego, CA) after each dog was sampled. Three superficial skin swabs (Isohelix, Cell Projects Ltd, UK) were used for each body site, with swabs being rubbed 10 times on each side of the swab within an area of approximately one square inch. Two swabs were immediately stored in lysis buffer from the MoBio Power Soil DNA Extraction kit (MoBio Laboratories, Inc., CA) to be extracted and sent for sequencing, and the third was retained in a sterile tube for archiving purposes. All swabs were stored at 4°C for no more than 1 week before extraction and final storage at –80°C. DNA was isolated using the MoBio Power Soil DNA Extraction kit, following the manufacturer's protocol. Negative controls were also included in sequencing: including sterile swabs that were processed following the extraction protocol, and the reagents only, with no swab included.

For comparison of sequences between our extraction protocol and the protocol followed by Findley *et al*. (2013), we collected four swabs from the right ear of five healthy dogs taking the same precautions as above. Two swabs were used following the above DNA extraction protocol and two followed the Findley *et al*. (2013) protocol.

### ITS sequencing and sequence analysis

Extracted DNA was submitted to MR DNA Laboratory (Shallowater, TX) for Illumina sequencing on a MiSeq Instrument using ITS1F (5′-CTTGGTCATTTAGAGGAAGTAA-3′) and ITS4R (5′-TCCTCCGCTTATTGATATGC-3′) primers. Resultant sequences from the forward reads were processed in Mothur (Schloss *et al*. 2009), an open-source bioinformatics software. First, sequences were trimmed for quality, sequences less than 200 bases were culled out and the remaining were chopped at 250 bases. Next, chimeras were removed with Uchime (Edgar *et al*. 2011), and OTUs were binned by taxonomic classification (phylotype) with the ITS-1 (Findley *et al*. 2013) database following their recommended parameters. Alpha diversities including inverse Simpson, non-parametric Shannon, Chao1, and observed species were calculated with a rarefaction depth of 1900 sequences. Distance matrices were generated using Bray–Curtis, Jaccard and Yue–Clayton theta coefficient metrics, with a rarefaction to 1900 sequences. These distance matrices were formatted for use within QIIME to generate PCoA plots.

### Statistical analysis

Alpha diversity estimators and relative abundances were first confirmed non-normal with the Shapiro–Wilk test in the statistical software JMP Pro 11 (SAS Institute, Inc.). A significance value of *P* < 0.05 was selected for all statistical tests. A Kruskal–Wallis test was performed to determine if the alpha diversity of at least one body site or dog was significantly different from the others. When significance was identified, a Steel–Dwass All Pairs test was performed to identify the body sites or dogs that were significantly increased or decreased (JMP). A Mann–Whitney test was performed for each shared body site (a body site that was sampled in both healthy and allergic dogs; *n* = 6), to determine whether the samples for one health status were significantly different from the other (JMP). Analysis of similarities (ANOSIM) function in the statistical software package PRIMER 6 (PRIMER-E Ltd, Luton, UK) was performed on Mothur-generated distance matrices to determine the influence of various factors (body site, individual dog, health status) on the dissimilarity between mycobiota of the groups being examined. The relative abundance tables generated in Mothur for each taxonomic level were combined and filtered to only include taxa that were present in at least 20 samples at greater than or equal to 0.1%. To identify taxa whose relative abundance was significantly different between body sites, individual dogs or health statuses, the filtered relative abundance table was imported into JMP and Kruskal–Wallis tests were performed. The filtered relative abundance table was also formatted for linear discriminant analysis (LDA) effect size (LEfSe) (Segata *et al*. 2011) to identify significant differences in taxa between health statuses. All *P* values were corrected for multiple comparisons using the Benjamini and Hochberg False discovery rate (Hochberg and Benjamini 1990).

## RESULTS

From the 148 canine body sites sampled, four were removed from analysis due to low number of sequences. The total number of fungal sequences amplified from the remaining 144 samples totaled 4 477 229 after quality processing and chimera removal; the median number of sequences per sample was 30 354.

### Fungal diversity analyses of healthy canine skin

**Figure 1.**
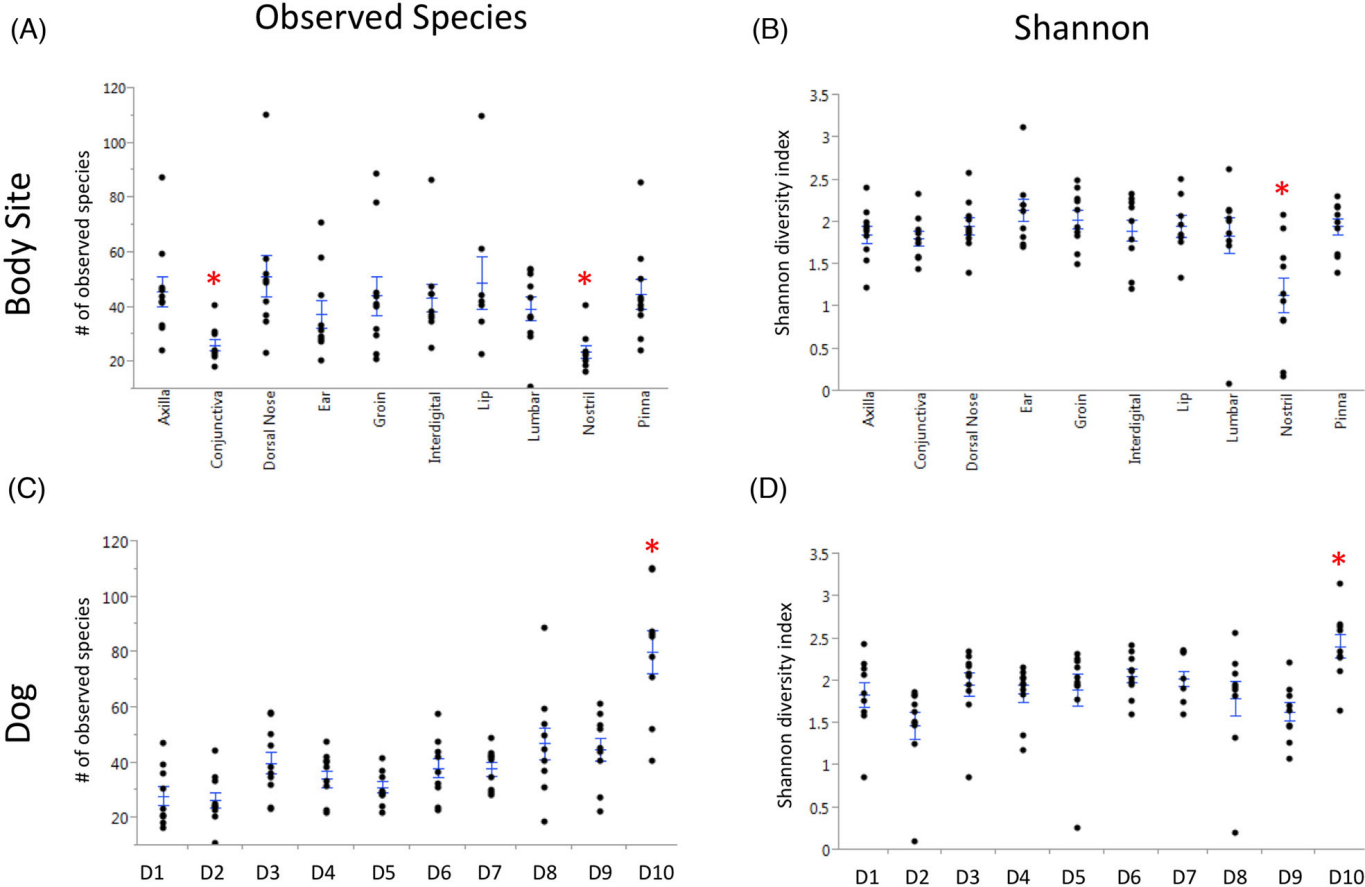
Alpha diversity of healthy dogs. Means are marked with straight line and mean error bars plotted using JMP. Asterisks denote body sites that are significantly different from all other sites (Kruskal–Wallis, multiple comparisons test, *P* < 0.05). (**a**) Species richness estimator was calculated with observed species and samples were grouped by body site. (**b**) Diversity estimator was calculated with Shan**no**n, and samples were grouped by body site. (**c**) Species richness estimator was calculated with observed species and samples were grouped by dog. (**d**) Diversity estimator was calculated with Shannon, and samples were grouped by dog.

Two factors were considered in the diversity analyses of healthy dogs: the influence of body site and of the dog. To test the effect of body sites, the same sites from all dogs were analyzed as a group. Conversely, to test the effect of the dog, all body sites from the same dog were analyzed as a group. Next, the diversity estimators for each group were compared. If there were significant differences between the groups, we concluded that factor had an influence on the diversity of the cutaneous mycobiota. We found an overall significant effect of body site on the richness (observed species, *P* = 0.0002; Table S1, Supporting Information) and diversity (Shannon, *P* = 0.028; Table S1, Supporting Information) of the cutaneous mycobiota. The mucosal surfaces, nostril and conjunctiva, accounted for most of this difference and had a significantly reduced number of observed species compared to all other sites (*P* < 0.05; Fig. 1a). However, when taking into account evenness with the Shannon metric, we found that only the nostril was significantly less diverse (*P* < 0.05; Fig. 1b) than all other sites. We also found a significant effect of the individual dog on the richness (observed species *P* < 0.0001; Table S1, Supporting Information) and diversity (Shannon *P* = 0.0003; Table S1, Supporting Information) of mycobiota. The mycobiota of dog number 10 was more rich (*P* < 0.05; Fig. 1c) and diverse (*P* < 0.05; Fig. 1d) than that of all other dogs. Median values of alpha diversity for each body site and dog are reported in Tables S2 and S3 (Supporting Information).

The same two factors were examined to determine their influence on the membership and structure of fungal communities (beta diversity) residing on healthy canine skin. To answer this question, ANOSIM was performed on rarefied distance matrices (membership: Bray–Curtis and Jaccard metrics; structure: Yue–Clayton coefficient). To determine the effect of one factor on the membership or structure, pairwise comparisons were made between all body sites or between all dogs. An *R* value and *P* value were produced for each comparison, and an *R* value closer to zero indicated similarity between the pair, whereas an *R* value closer to one indicated dissimilarity between the pair. When examining a factor, higher *R* values indicated that factor has an influence on the beta diversity.

**Figure 2.**
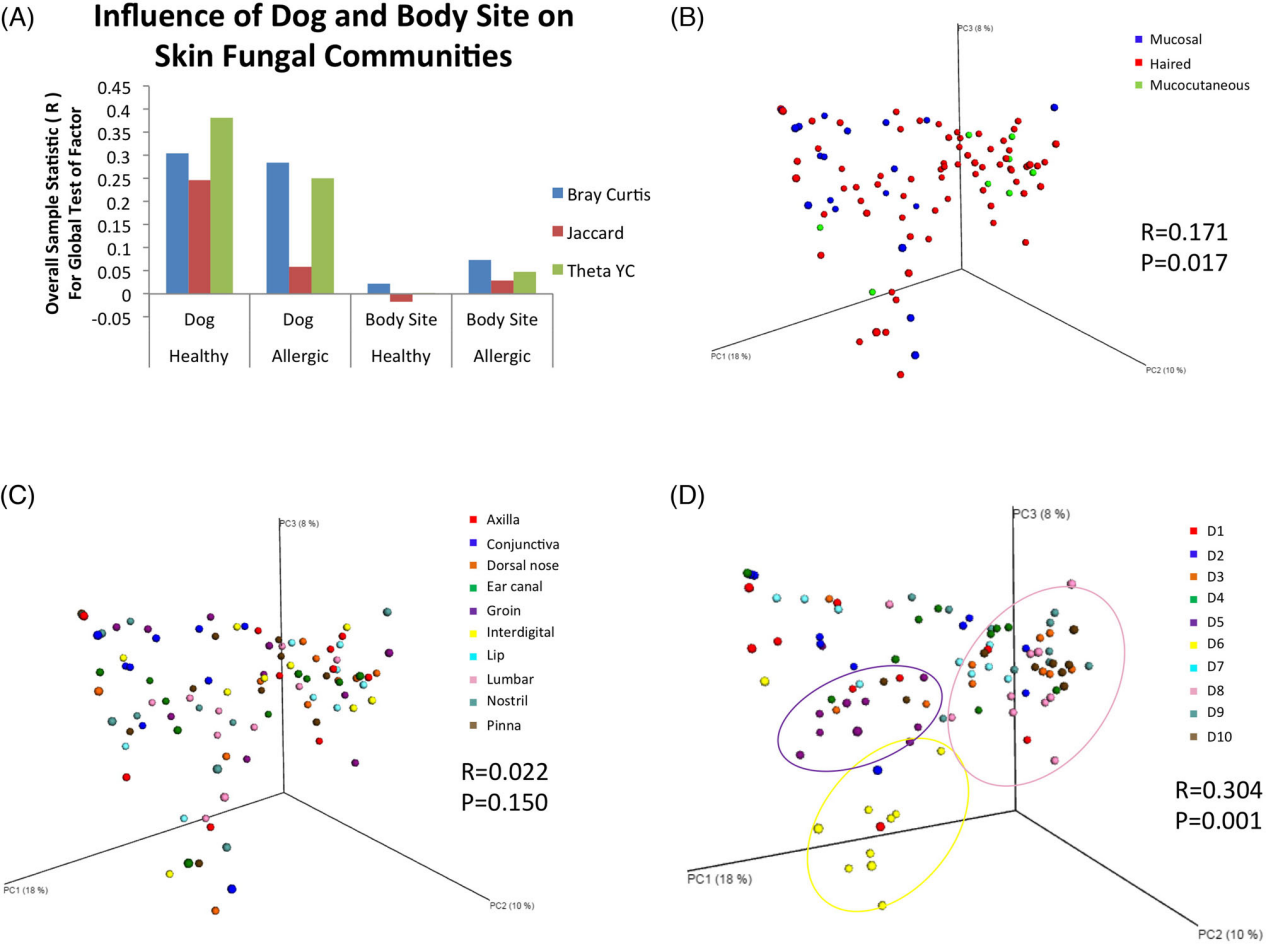
The influence of skin microenvironment, body site and dog on fungal community membership. (**a**) The overall sample statistic (R) from the global test for the two factors ‘Body Site’ and ‘Dog’ were calculated in Primer6 with ANOSIM on the distance matrix containing only healthy or only allergic dogs generated in Mothur with the following metrics: Bray–Curtis (blue), Jaccard (red) and the Yue–Clayton theta coefficient (green). The *R* values for each test are plotted as bars and grouped by health status and factor tested. (**b**) PCoA plots were generated in QIIME with samples colored by skin microenvironment, and pairwise distance calculations were performed in Mothur using the Bray–Curtis metric. (**c**) PCoA plots were generated in QIIME with samples colored by body site, and pairwise distance calculations were performed in Mothur using the Bray–Curtis metric. (**d**) PCoA plots were generated in QIIME with samples colored by dog, and pairwise distance calculations were performed in Mothur using the Bray–Curtis metric.

We found that the individual dog factor had a greater influence (median *R* values = 0.338, 0.535, 0.381, respectively; Table S4) on the beta diversity of cutaneous mycobiota than did the body site factor, which was not a significant influencing factor for any of the 45 pairwise comparisons made between healthy body sites (Fig. 2a). From the 45 comparisons between healthy dogs, 38 pairs were significantly dissimilar (*P* < 0.05; Table S4, Supporting Information). These findings were visualized with PCoA plots generated from rarefied Bray–Curtis distance matrices, which showed no sample clustering by skin microenvironment (Fig. 2b), or body site (Fig. 2c), but showed clustering of all body sites for several dogs (Fig. 2d). However, not every dog demonstrated tight clustering of body sites.

### Fungal community composition of healthy canine skin

**Figure 3.**
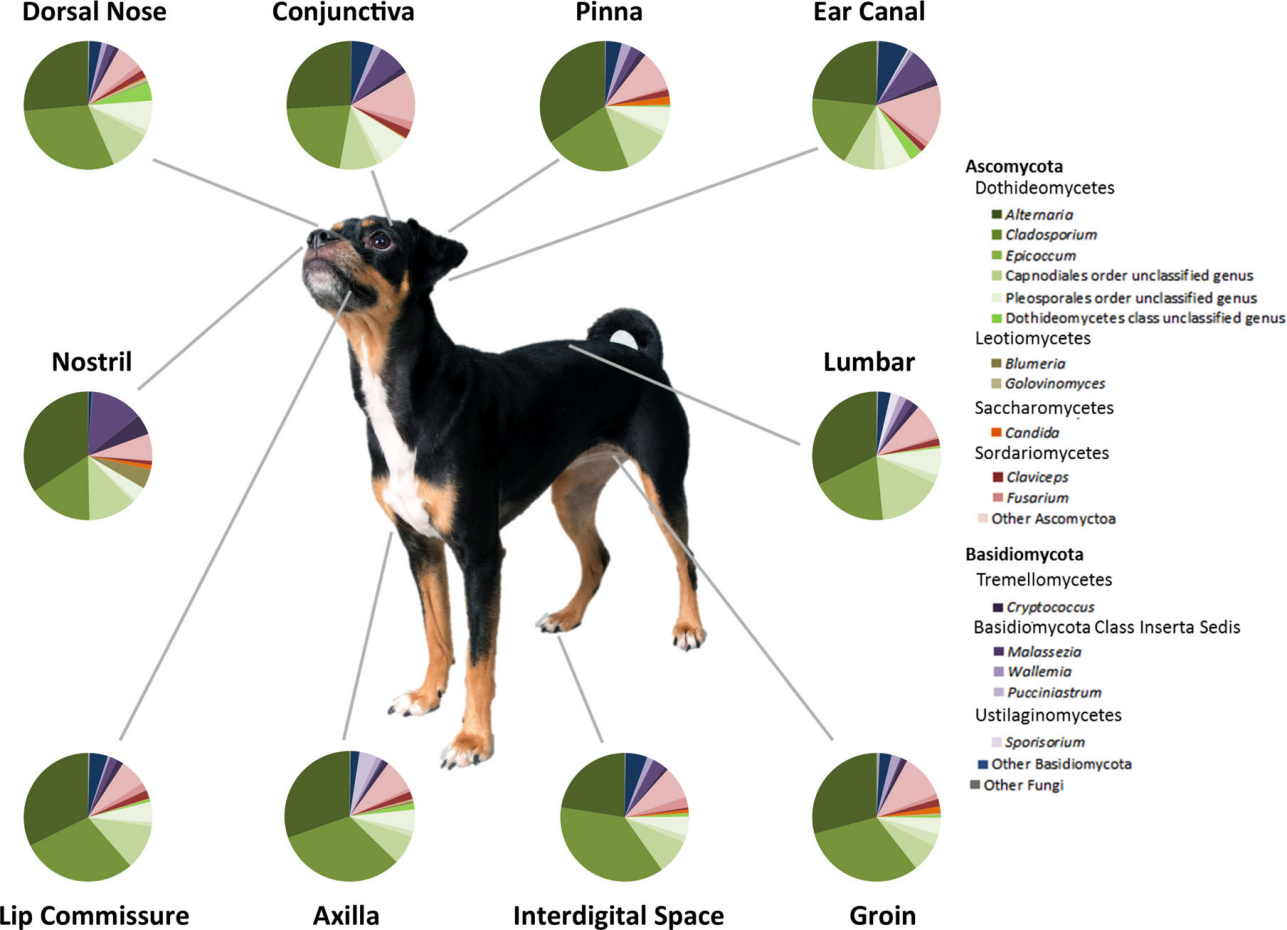
Average relative abundance of fungal taxa by body site in healthy dogs. The average relative abundance of predominant taxa was calculated for each body site and represented by pie charts. The averages were taken from D1-10.

In addition to diversity analyses, the taxonomic composition of the mycobiota was also determined. The predominant phylum of fungal organisms sequenced from healthy canine skin was Ascomycota followed by Basidiomycota. The major class of Ascomycetes was Dothideomycetes and the most abundant genera within this class included *Alternaria*, *Cladosporium* and *Epicoccum*. The most abundant Basidiomycete genera included *Cryptococcus* and *Malassezia*. There were also other Ascomycete taxa that were abundant but unable to be classified to the genus level based on available fungal databases (Fig. 3).

**Figure 4.**
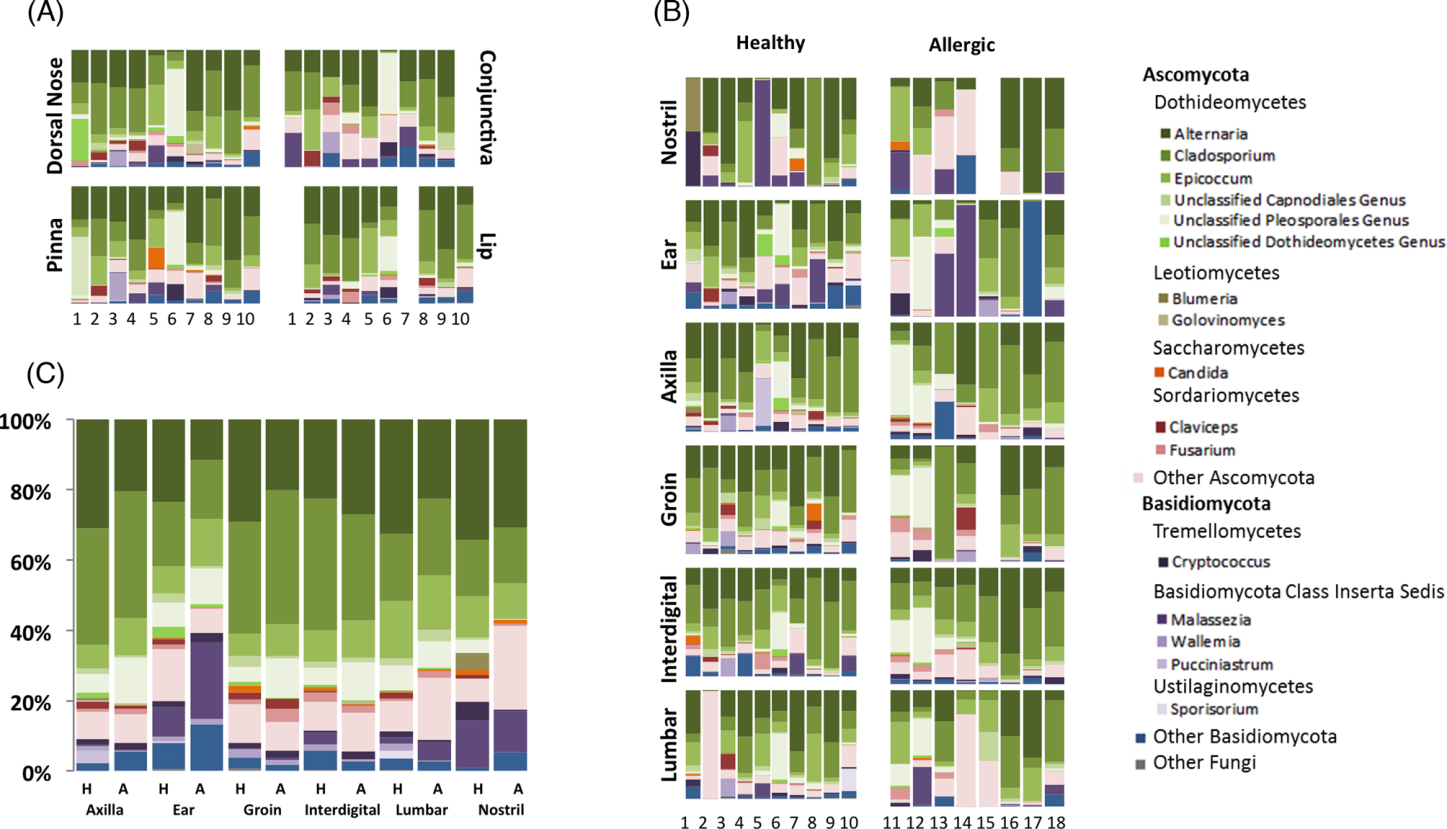
Fungal taxa summary plots for healthy and allergic canine skin. Stacked bar plots represent the predominant fungal taxa present within a sample. (**a**) Body sites are arranged in rows with each column representing the body site of one dog (numbered at the bottom). (**b**) Shared sites between healthy and allergic dogs are arranged with healthy on the left and allergic on the right in a similar orientation as (a). (**c**) The relative abundance of predominant fungal taxa was averaged across all dogs in each health status group for each body site. H represents healthy dogs and A represents allergic.

To determine whether the relative abundance of specific fungal taxa differed between body sites or individual dogs, statistical analysis was performed on the relative abundance tables generated from Mothur. The relative abundance table, including all taxonomic levels, was filtered to only include taxa that were present in at least 20 samples at greater than 0.1%. Using this filtered table that included 193 taxa, we identified four fungal taxa that were significantly different between body sites in healthy dogs, and 153 that were significantly different between dogs (*P* < 0.05; Table S5, Supporting Information). The LEfSe analysis did not reveal any taxa that were significantly different between body sites. These findings are further visualized by stacked bar plots of the relative abundances of fungal taxa for each sample, which showed a high degree of variation between dogs (columns) for the same body site (rows) (Fig. 4a and b). Similar to the diversity analysis, the individual dog factor had a greater influence on community composition than did the body site factor, indicating that specific taxa were found across all body sites in one dog, but not present on other dogs.

To test if the extraction protocol used in this study had an effect on the ability to extract *Malassezia* DNA, we compared this extraction protocol to that of Findley *et al*. (2013). Comparison of the two extraction protocols yielded no significant differences in the relative abundance of *Malassezia* detected in the ears of five dogs (Fig. S1a, Supporting Information), and both protocols yielded similar most abundant taxa: *Cladosporium* and *Epiccocum* (Fig. S1b, Supporting Information). However, there did exist an influence of protocol on the data, as relative proportions of *Cladosporium* and *Epiccocum* varied between the two protocols, samples clustered separately by protocol on PCoA, and overall community membership and structure were significantly different (ANOSIM *R* = 0.3880, *P* = 0.0370; Fig. S1c, Supporting Information).

### Fungal diversity analyses of baseline allergic canine skin

Similar to the approach in healthy dogs, we were also interested in how body site or dog influenced the mycobiota of allergic skin in dogs. There was an overall significant effect of body site on richness (observed species, *P* < 0.0001) and diversity (Shannon, *P* = 0.030) of cutaneous mycobiota (Table S1, Supporting Information). Specifically the nostril was less rich (observed species, *P* < 0.01) than the axilla, groin, interdigital and lumbar sites, and less diverse (Shannon, *P* < 0.05) than the axilla, groin and interdigital sites. In addition, the ear in allergic dogs was both less rich (observed species, *P* < 0.05) than the axilla, groin and lumbar and less diverse (Shannon, *P* < 0.05) than the interdigital space. In contrast to the findings in healthy dogs, there was no influence of the individual dog on the richness or diversity of the skin mycobiota. Exact values of alpha diversity for each body site and dog are reported in Tables S6 and S7 (Supporting Information).

Although there were no significant differences in beta diversity of cutaneous mycobiota between body sites in healthy dogs, differences between body sites in allergic dogs were identified. Fungal community membership (Bray–Curtis) was significantly different in the nostril compared to the axilla (*R* = 0.331; *P* = 0.015; Table S8, Supporting Information) and interdigital space (*R* = 0.441; *P* = 0.015; Table S8, Supporting Information). Fungal community structure (Yue–Clayton theta coefficient) was different between the nostril and axilla (*R* = 0.294; *P* = 0.030; Table S8, Supporting Information), groin (*R* = 0.213; *P* = 0.030; Table S8, Supporting Information) and interdigital space (*R* = 0.300; *P* = 0.045; Table S8, Supporting Information). Similar to healthy dogs, the beta diversity was more dependent on the individual dog than body site (Fig. 2a) with 11 out of 28 comparisons between allergic dogs being significantly different with a median *R* value of 0.306 for membership (Bray–Curtis, median *P* < 0.05; Table S4, Supporting Information) and 0.297 for structure (Yue–Clayton theta coefficient, median *P* < 0.05; Table S4, Supporting Information).

### Shifts in attributes of cutaneous mycobiota between healthy and allergic dogs

**Figure 5.**
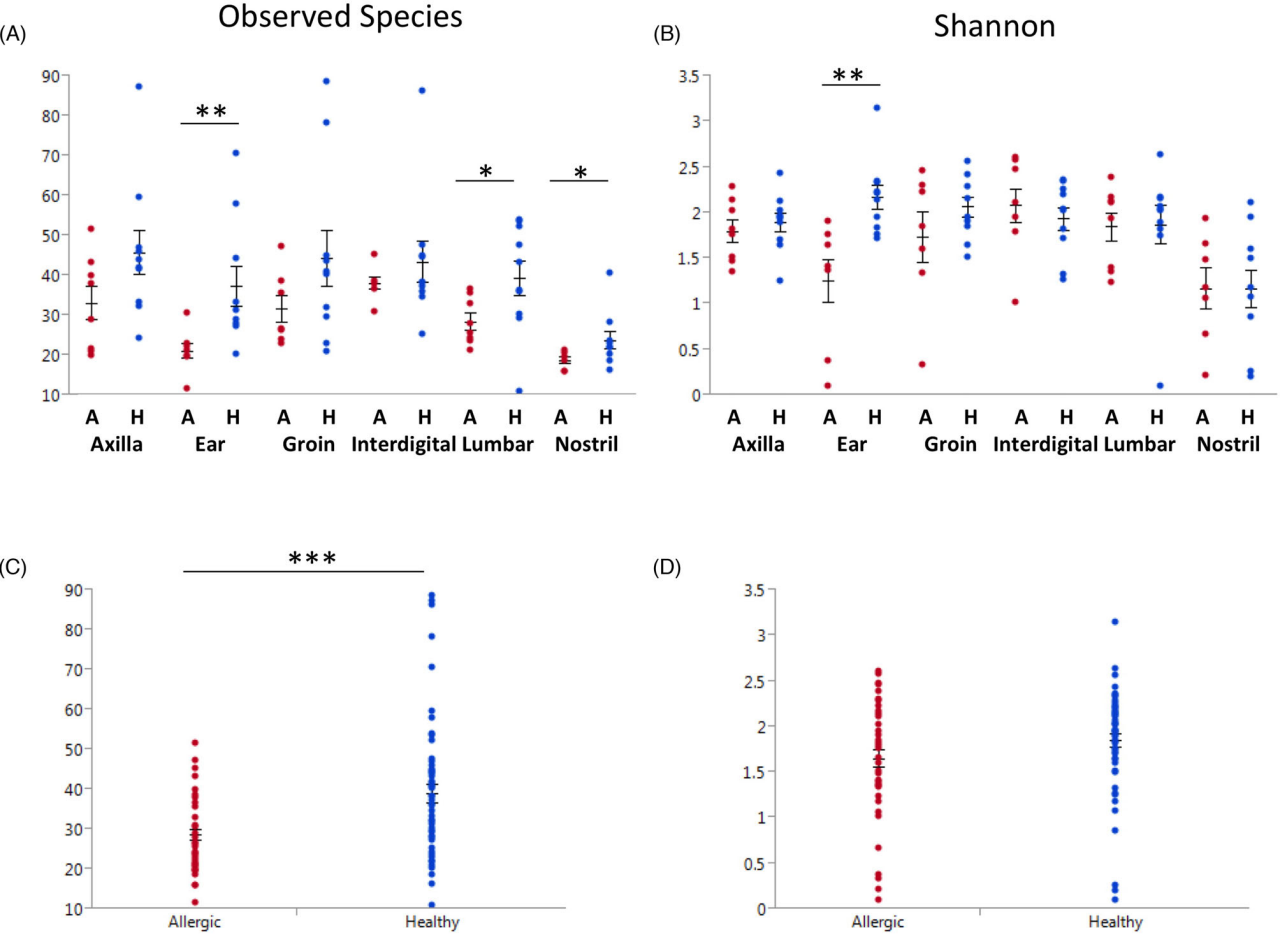
Comparison of alpha diversity and richness between healthy and allergic dogs. Means are marked with straight lines and mean error bars plotted using JMP. Significant differences between health statuses are denoted by asterisks (*P < 0.05, **P < 0.01, ***P < 0.001). (**a**) The fungal richness of each body site was calculated with observed species, and are grouped by body sited and health status. (**b**) The fungal diversity of each body site was calculated with Shannon, and are grouped by body sited and health status. (**c**) The fungal richness of body sites was calculated with observed species, and are grouped by health status. (**d**) The fungal diversity of body sites was calculated with Shannon, and are grouped by health status.

For the comparison of mycobiota between healthy and allergic canine skin, only the ‘health status’ was considered. First, differences in alpha diversity between the two groups were evaluated. The mycobiota of the nostril and ear from allergic dogs were less rich than their counterpart in healthy dogs (observed species, *P* < 0.05 and *P* < 0.01; Fig. 5a). The mycobiota of the ear was the only allergic body site that was less diverse (Shannon, *P* = 0.003; Fig. 5b) than the same body site in healthy dogs. Overall allergic canine skin was significantly less rich in fungal species compared to healthy canine skin (observed species, *P* < 0.001; Fig. 5c), but not significantly different for the evenness measurements (Fig. 5d).

**Figure 6.**
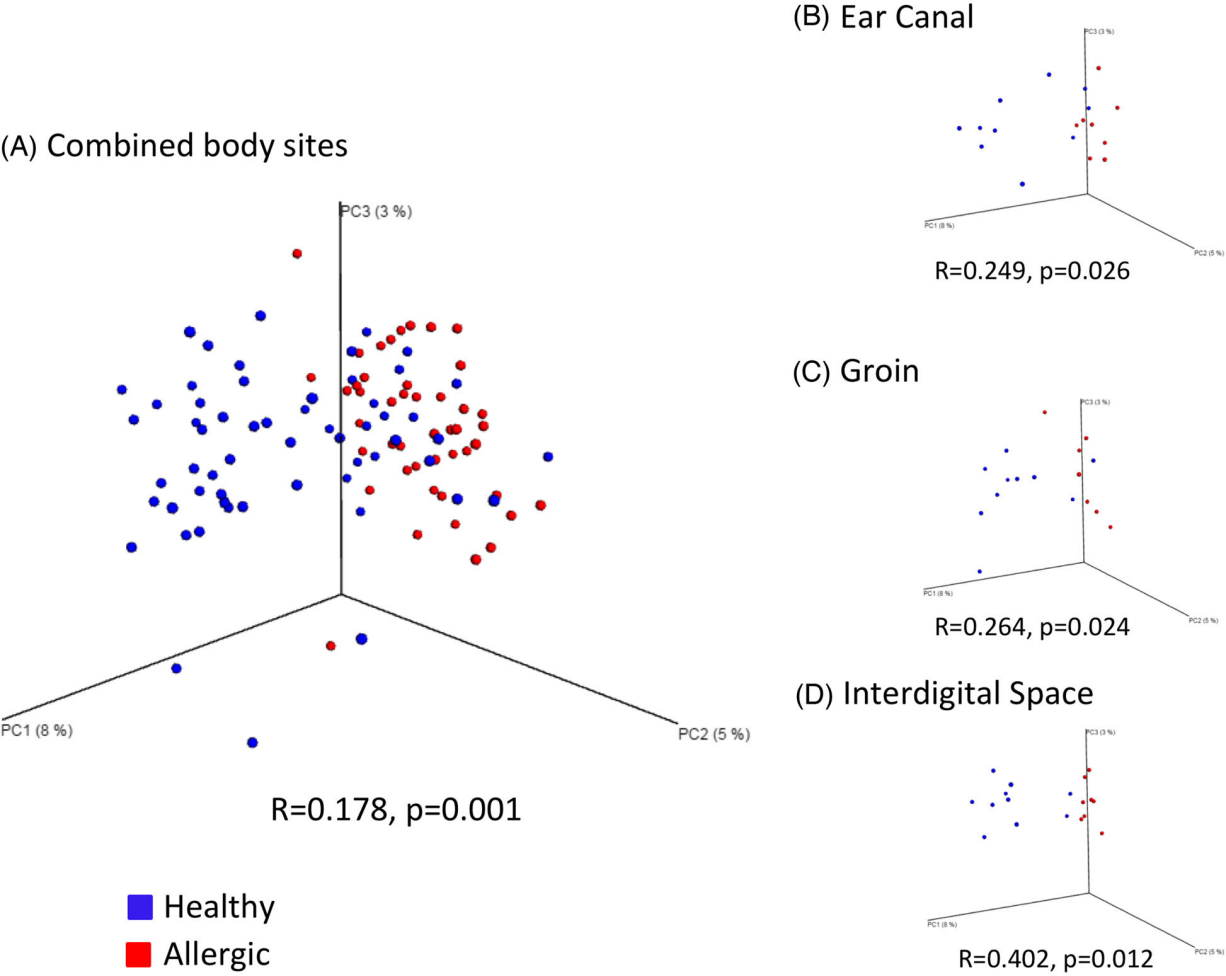
Dissimilarity between healthy and allergic fungal communities. (**a**) PCoA plot of all samples coming from the shared sites in healthy and allergic dogs. Difference in fungal community membership was estimated with the Jaccard metric in the Mothur package, and 3-D PCoA plots were generated in QIIME. Each dot represents a body site from one dog, with all healthy dogs colored in blue and all allergic dogs colored in red. (**b**) PCoA plot of only the ear samples for healthy and allergic dogs. (**c**) PCoA plot of only the groin samples for healthy and allergic dogs. (**d**) PCoA plot of only the interdigital space samples for healthy and allergic dogs.

**Figure 7.**
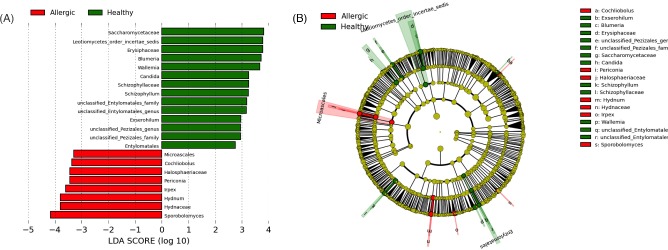
Differential abundances of fungal taxa between healthy and allergic canine skin. (**a**) LEfSe analysis revealed 14 fungal taxa significantly more abundant in healthy skin, and 8 taxa more abundant in allergic skin. (**b**) Cladogram plotted from LEfSe analysis showing the taxonomic levels represented by rings with phyla in the innermost ring and genera in the outermost ring, and each circle is a member within that level. Those taxa in each level are colored by health status for which it is more abundant (*P* < 0.05; LDA score 2.5).

We also wanted to know whether health status influenced beta diversity. To accomplish this, ANOSIM was performed on the Jaccard distance matrix and an overall significant effect of the factor ‘health status’ was identified (Fig. 6a), which was mainly due to differences in cutaneous mycobiota between health status groups at three body sites: ear canal (*R* = 0.249, *P* = 0.026; Table S8, Supporting Information), groin (*R* = 0.264, *P* = 0.024; Table S8, Supporting Information), and interdigital space (*R* = 0.402, *P* = 0.012; Table S8, Supporting Information). These differences were visualized with PCoA plots that demonstrated clear clustering of allergic dog samples separate from healthy dog samples for individual body sites (Fig. 6b–d). These findings were further supported by testing of individual taxa between the two health status groups done in JMP using the filtered relative abundance table (Table S5, Supporting Information), and through LEfSe analysis (Fig. 7).

In addition to alpha- and beta diversity, differences in relative abundance of specific taxa were also identified between healthy and allergic dogs. Overall, there were 85 taxa found to be significantly increased or decreased in allergic skin when compared with healthy skin by the Kruskal–Wallis test performed in JMP (*P* < 0.05; Table S5, Supporting Information). Stacked bar plots were used to visualize changes in presence and abundance of fungal taxa between healthy and allergic skin at six body sites (Fig. 4b and c). LEfSe analysis identified 12 taxa that were more abundant in healthy dogs, and 7 taxa that were more abundant in allergic dogs (Fig. 7). The genera that were increased in healthy skin included *Blumeria*, *Wallemia*, *Candida*, *Schizophyllum* and *Exserhilum*. The genera increased in allergic skin include *Sporobolomyces*, *Hydnum*, *Irpex*, *Periconia*, *Cochliobolus* and *Microascales*. Furthermore, 50% of the allergic ears were predominated by one genus: 58% of the mycobiota in the ear of D13 was *Malassezia*, 94% of D14 was *Malassezia* and 99% of D17 was *Sporobolomyces* (Basidiomycete, Sporidiobolales family *incerta sedis*) (Fig. 4b).

## DISCUSSION

Consistently throughout the diversity analyses, and comparison of relative abundances of fungal taxa, cutaneous mycobiota of 10 dogs sampled in this study were more dependent on the individual dog than the body site. Although the mycobiota associated with body sites were very similar within the dog, there existed a high degree of inter-dog variability. Human skin also exhibits a high degree of interpersonal variability (Findley *et al*. 2013), but unlike canine skin, as demonstrated by the current study, human cutaneous mycobiota were dependent on body site. Although body site was not a major influencing factor of cutaneous mycobiota in healthy dogs, we did find reduced fungal diversity at the mucosal sites. The mucosal sites in dogs, including the nostril and conjunctiva, are bathed in fluid and are also more protected from the environment, which could explain the reduction in richness, a finding that also exists for the bacterial microbiota of canine skin (Rodrigues Hoffmann *et al*. 2014).

Several physiological differences exist between canine and human skin which may account for some of the differences noted between the mycobiota of the two species. Human skin has a varied topography and morphology, producing distinct dry, sebaceous or moist skin microenvironments (Grice and Segre 2011), whereas canine skin is more uniform across areas of haired skin containing both sebaceous and apocrine glands (Miller *et al*. 2013a). Additionally, canine skin is more acidic than human skin (Matousek and Campbell 2002; Oh and Oh 2010). There also exists differences in lipid content of the skin that could influence the colonization of cutaneous mycobiota (Miller *et al*. 2013a).

Other than skin morphology and topography, additional factors may be responsible for the differences in distribution of mycobiota observed between canine and human skin. These include differences between human and animal behavior, hygiene habits and amount of environmental exposure. It is generally well accepted that dogs are more exposed to outdoor elements than humans due to closer proximity to the ground, and behaviors such as rolling in the grass and laying on the floor inside the home where shoes track in environmental contaminants. In addition to greater environmental exposure, dogs are bathed less frequently than humans, which could enable colonization of more diverse fungi.

The types of environmental exposure could affect beta diversity of cutaneous mycobiota, which may explain the high degree of inter-dog variability. For example, dogs from different homes (and backyards) may have variable exposures to different types of trees, plants, grasses and bodies of water, such as ponds, swimming pools or bayous. Contact with the floor inside a home is another type of environmental exposure that likely influences diversity. It is interesting, and possibly correlated, that the only areas of human skin possessing high fungal diversity are the feet (Findley *et al*. 2013), which are often in contact with the floor. Similarly, dogs spend most of their time laying their entire body on the floor, and a diverse mycobiota, as identified in this study, would be expected to colonize different regions of their bodies. Additional indoor exposures, including cohabitation with other people or animals, can also influence the cutaneous microbiota (Song *et al*. 2013). Further studies are required to find a true correlation between cohabitation and sharing of cutaneous mycobiota amongst human and animal members of the same household.

We also found that health status had a significant effect on the cutaneous mycobiota of dogs. Comparing 10 healthy dogs in this study with 8 dogs diagnosed with allergic skin disease, we found that the skin of allergic dogs had reduced fungal richness. Additionally, allergic ears, a site commonly infected in allergic dogs, had reduced fungal diversity as well. Interestingly, we identified differences in cutaneous mycobiota between body sites in allergic dogs, but not in healthy dogs. Also, the cutaneous mycobiota was more similar within a dog for healthy dogs than for allergic dogs. These findings taken together suggest that the stability of cutaneous mycobiota within a dog is disturbed by allergic skin disease, leading to changes at distinct body sites affected by this disease, and thus more pronounced body site differences in the allergic dogs. A similar phenomenon was observed for human primary immunodeficiency patients where the dependence of bacterial communities on body site in healthy individuals was diminished in the affected individuals (Oh *et al*. 2013).

The significant changes identified for allergic skin in dogs who were not experiencing any observable clinical lesions at the time of sample collection suggest an association of fungal dysbiosis to the underlying mechanisms of allergic skin disease. In people, loss-of-function mutations to the filaggrin gene and resultant skin barrier dysfunction have been proposed as one of the most important factors in development of AD (Palmer *et al*. 2006). Altered filaggrin expression has been identified for atopic dogs (Santoro *et al*. 2013), along with transepidermal water loss, and decreased ceramide concentrations (Shimada *et al*. 2009). It is possible that the reduced diversity we see in both bacteria and fungi living on non-lesional allergic canine skin could be attributed to skin barrier impairment in allergic dogs, or to changes in nutrient (water and lipid) availability in the skin caused by the allergic skin disease. Likewise, the chronic use of steroids, antibiotics, antifungals, fatty acids and topical treatments in allergic dogs could alter the skin microbiota.

A previous study reported an increase in fungal diversity in lesional skin of atopic people (Oh *et al*. 2013). Although we did not find the same trend in our study, this may be attributed to the fact that the mycobiome of healthy human skin is naturally less diverse than healthy canine skin, and predominated by one genus, *Malassezia*. The healthy human vaginal microbiota lacks bacterial diversity and is predominated by *Lactobacillus* (Fredricks, Fiedler and Marrazzo 2005; Ma, Forney and Ravel 2012). An increased diversity of anaerobic bacteria, coupled with a decrease in *Lactobacillus*, has been identified in bacterial vaginosis (BV) through NGS (Fredricks, Fiedler and Marrazzo 2005; Ma, Forney and Ravel 2012). In both of these cases of increased microbial diversity associated with diseased skin and mucosa (fungal microbiota in AD and bacterial microbiota in BV), the baseline or healthy microbiota is predominated by one genus, and a disturbance to the microbiota lead to a decrease of the major microbial resident allowing for invasion of other microbes, thus an increase in overall diversity. On the other hand, a disruption to an already diverse microbiota could allow for one or several microbes to increase in relative abundances, and predominate in lesional skin. Another possibility is that decreased microbial diversity was present in non-lesional canine skin, but had lesional skin been sampled, an increase in diversity might have been observed. We plan to evaluate the differences between lesional and non-lesional canine allergic skin in future studies.

Although *Malassezia* has been implicated in both human and canine AD as an allergen and trigger of disease symptoms (Morris, Olivier and Rosser 1998; Bond *et al*. 2006; Casagrande *et al*. 2006; Kato *et al*. 2006; Zhang *et al*. 2011a), we were unable to detect any significant differences in the relative abundance of *Malassezia* between healthy and allergic groups. Three ears were predominated by *Malassezia* (greater than 50% relative abundance), one from a healthy dog and two from allergic dogs. All dogs were examined by veterinarians and there were no reported ear infections at the time of sample collection; thus, these would either represent asymptomatic ear infections or the invasion and predominance of one genus. Perhaps if more dogs had been sampled we would have seen a true significant increase in *Malassezia* for the allergic group. The relatively low abundance of *Malassezia* across all body sites was an unexpected finding since culture-based studies have reported *Malassezia* as being one of the most cultured fungi from canine skin (Bensignor *et al*. 2002; Prado *et al*. 2008; Brito *et al*. 2009; Campbell *et al*. 2010). This genus absolutely predominated in human skin in two NGS studies targeting the ITS (Findley *et al*. 2013) and large rRNA subunit regions (Zhang *et al*. 2011b). We ruled out the possibility of the extraction protocol influencing the amount of *Malassezia* detected through comparison of our extraction protocol with that of the Findley *et al*. (2013) protocol, and found no significant differences in the amount of *Malassezia*.

Our results demonstrate a very rich mycobiome compared to human skin with a predominance of Dothideomycetes such as *Alternaria*, *Cladosporium* and *Epicoccum*. These three genera are responsible for environmental allergies in two groups of people: 20%–30% of atopic people, and 6% of the general population (non-atopic people) (Horner *et al*. 1995). In addition to serving as human allergens to hypersensitive people, these fungi are also known allergens for atopic dogs (Kang *et al*. 2014), and have been identified in house dust (Fujimura *et al*. 2010). Interestingly, the relative abundances of *Alternaria* and *Cladosporium* were significantly different between dogs (Fig. S3, Supporting Information) and future studies may help to elucidate why the skin of some dogs harbor more of these allergenic fungi than do others, and whether carriage of these fungi on dogs could impact humans or dogs who are hypersensitive to these fungi. Furthermore, it is possible that cohabitation with dogs, whose skin is inhabited by these allergenic fungi, at an early age could desensitize children to fungal allergens and possibly protect against the development of allergies to these fungi (Wegienka *et al*. 2011). Another possible mechanism for desensitization to the allergenic fungi present on the coat of dogs is through fetal exposure in a pregnant mother who cohabits with dogs (Aichbhaumik *et al*. 2008; Havstad *et al*. 2011).

In summary, NGS of canine skin has revealed a much more diverse cutaneous mycobiota than what was previously described with culture-based techniques. The cutaneous mycobiota appear to be influenced by various factors including environmental exposure, cohabitation with other pets and skin health status. Since the majority of the dogs in our study came from separate households and were different breeds, the high degree of inter-dog variability could be explained by differences in environmental exposure, genetic differences between breeds or pelage characteristics. Our study only included 18 dogs and so the influence of these factors should be confirmed with future studies having increased numbers of animals and evaluating each factor separately. The host–microbiome interaction in allergic dogs also warrants investigation through immunologic and metagenomic studies, as we have now seen both the bacterial and fungal microbiota in non-lesional canine allergic skin disease are disrupted, with increased abundances of particular taxa present in the allergic skin, and an overall reduction in microbial diversity. The predominant fungal taxa inhabiting the skin of dogs suggest human cohabitation with dogs could have an effect on sensitization to fungi, and other microbes; however, this relationship and mechanism remain unclear.

## Supplementary Material

Supplementary data are available at FEMSEC online
