# Supplementary material for: What is living on your dog's skin? Characterization of the canine cutaneous mycobiota and fungal dysbiosis in canine allergic dermatitis
Source: FEMS Microbiol Ecol. 2015 Nov 5;91(12):fiv139. doi: 10.1093/femsec/fiv139 (PMC4657189; doi:10.1093/femsec/fiv139)
Supplement: Supplementary data are available at FEMSEC online [file Supplement_Materials.zip › Supplementary Tables.docx]

Supplementary Table S1. Overall effect of body site, dog, and health status on alpha diversity.

| **Group** | **Factor** | **Observed Species** | **Chao1** | **Shannon** | **Inverse Simpson** |
| --- | --- | --- | --- | --- | --- |
| Healthy | Body Site | 0.0002 | 0.0004 | 0.0283 | 0.1380 |
|  | Dog | <0.0001 | <0.0001 | 0.0003 | 0.0088 |
| Allergic | Body Site | <0.0001 | 0.0030 | 0.0297 | 0.1657 |
|  | Dog | 0.9480 | 0.8437 | 0.0900 | 0.0455 |
| Shared Sites | Health Status | 0.0005 | 0.0047 | 0.0807 | 0.3965 |

Supplementary Table S2. Alpha diversity calculations by site in healthy skin group.

| **Skin Site** | **Observed Species** | | **Chao1** | | **Shannon** | | **Inverse Simpson** | |
| --- | --- | --- | --- | --- | --- | --- | --- | --- |
|  | Median | Min-Max | Median | Min-Max | Median | Min-Max | Median | Min-Max |
| Axilla | 46 | 24-87 | 66 | 40-140 | 1.9 | 1.3-2.4 | 1.9 | 2.1-6.4 |
| Conjunctiva | 26 | 18-41 | 40 | 29-55 | 1.8 | 1.5-2.3 | 1.8 | 3.1-6.8 |
| Dorsal Nose | 51 | 23-110 | 75 | 40-157 | 2.0 | 1.5-2.7 | 2.0 | 2.6-5.7 |
| Ear | 37 | 20-71 | 52 | 32-88 | 2.2 | 1.7-3.1 | 2.2 | 3.3-10.0 |
| Groin | 44 | 21-89 | 63 | 36-135 | 2.1 | 1.5-2.6 | 2.1 | 3.0-6.8 |
| Interdigital | 43 | 25-86 | 64 | 40-116 | 1.9 | 1.3-2.4 | 1.9 | 1.9-7.0 |
| Lip | 49 | 23-110 | 68 | 40-151 | 2.0 | 1.4-2.6 | 2.0 | 2.9-6.6 |
| Lumbar | 39 | 11-54 | 56 | 19-73 | 1.9 | 0.1-2.6 | 1.9 | 1.0-8.5 |
| Nostril | 24 | 16-41 | 41 | 30-54 | 1.2 | 0.2-2.1 | 1.2 | 1.1-5.5 |
| Pinna | 45 | 24-86 | 67 | 40-146 | 2.0 | 1.5-2.3 | 2.0 | 2.3-7.5 |

Supplementary Table S3. Alpha diversity calculations by dog in healthy skin group.

| **Dog Number** | **Observed Species** | | **Chao1** | | **Shannon** | | **Inverse Simpson** | |
| --- | --- | --- | --- | --- | --- | --- | --- | --- |
|  | Median | Min-Max | Median | Min-Max | Median | Min-Max | Median | Min-Max |
| 1 | 23 | 16-47 | 40 | 29-63 | 1.8 | 0.9-2.4 | 4.7 | 2.1-7.6 |
| 2 | 24 | 11-44 | 40 | 19-58 | 1.6 | 0.1-1.9 | 3.7 | 1.0-4.5 |
| 3 | 37 | 23-58 | 56 | 38-79 | 2.1 | 0.9-2.4 | 4.8 | 1.6-6.7 |
| 4 | 36 | 22-48 | 51 | 34-70 | 2.0 | 1.2-2.2 | 4.1 | 2.5-5.4 |
| 5 | 29 | 22-42 | 47 | 41-65 | 2.0 | 0.3-2.3 | 5.1 | 1.1-7.5 |
| 6 | 39 | 23-58 | 53 | 32-87 | 2.1 | 1.6-2.4 | 4.9 | 3.0-6.8 |
| 7 | 41 | 28-49 | 53 | 42-66 | 2.0 | 1.6-2.4 | 3.5 | 2.9-7.2 |
| 8 | 45 | 19-89 | 61 | 43-135 | 1.9 | 0.2-2.6 | 3.8 | 1.1-6.3 |
| 9 | 45 | 22-62 | 64 | 38-85 | 1.7 | 1.1-2.2 | 3.0 | 1.8-5.3 |
| 10 | 86 | 41-110 | 116 | 54-157 | 2.3 | 1.6-3.1 | 4.8 | 2.1-10.0 |

Supplementary Table S4. Dissimilarity of fungal community membership and structure between dogs.

|  |  | **Healthy Dogs** | **Allergic Dogs** |
| --- | --- | --- | --- |
| **Bray Curtis** | Median R (min-max) | 0.338 (0.11-0.667) | 0.306 (0.176-0.559) |
|  | Median P value (FDR) (min-max) | 0.045 (0.003-0.049) | 0.023 (0.016-0.036) |
|  | Number of Significant Comparisons (total comparisons) | 38 (45) | 11 (28) |
| **Jaccard** | Median R (min-max) | 0.535 (0.147-0.836) | n/a |
|  | Median P value (FDR) (min-max) | 0.045 (0.006-0.049) | n/a |
|  | Number of Significant Comparisons (total comparisons) | 23 (45) | 0 |
| **Theta YC** | Median R (min-max) | 0.381 (0.108-0.954) | 0.297 (0.178-0.683) |
|  | Median P value (FDR) (min-max) | 0.045 (0.004-0.049) | 0.038 (0.019-0.04) |
|  | Number of Significant Comparisons (total comparisons) | 41 (45) | 8 (28) |

Supplementary Table S5. Combined and filtered relative abundance table for 193 fungal taxa that were tested for significant differences between body sites, dogs, and health status.

| **Phylum** | **Healthy (Dog)** | **Healthy (Body Site)** | **Allergic (Dog)** | **Allergic (Body Site)** | **Shared Sites (Health Status)** |
| --- | --- | --- | --- | --- | --- |
| **Class** |  |  |  |  |  |
| **Order** |  |  |  |  |  |
| **Family** |  |  |  |  |  |
| **Genus** |  |  |  |  |  |
| *Ascomycota* | **0.0012** | 0.5617 | 0.1749 | 0.4812 | 0.9898 |
| *Ascomycota_class_incertae_sedis* | **0.0350** | 0.4549 | 0.3241 | 0.6614 | 0.1631 |
| *Ascomycota_order_incertae_sedis* | **0.0379** | 0.5118 | 0.4102 | 0.7441 | 0.1398 |
| *Ascomycota_family_incertae_sedis* | **0.0305** | 0.5328 | 0.6147 | 0.7567 | 0.2343 |
| *Dothideomycetes* | **0.0290** | 0.4073 | 0.1037 | 0.6580 | 0.9746 |
| *Capnodiales* | **0.0000** | 0.1200 | 0.0543 | 0.5415 | 0.8457 |
| *Capnodiales_family_incertae_sedis* | **0.0031** | 0.0669 | 0.3477 | 0.5351 | 0.4063 |
| *Capnobotryella* | 0.6575 | 0.2633 | 0.9521 | 0.3389 | **0.0006** |
| *Cladosporium* | **0.0080** | 0.0562 | 0.2259 | 0.5272 | 0.3215 |
| *Dissoconium* | **0.0000** | 0.3329 | 0.6609 | 0.3989 | 0.2692 |
| *Ramichloridium* | **0.0010** | 0.7842 | 0.9718 | 0.3233 | **0.0095** |
| *Mycosphaerellaceae* | **0.0000** | 0.1038 | 0.1626 | 0.2639 | 0.7247 |
| *Cercospora* | **0.0001** | 0.7459 | 0.6544 | 0.3082 | 0.8293 |
| *Mycosphaerella* | **0.0027** | 0.3541 | 0.5455 | 0.3707 | **0.0014** |
| *unclassified_Mycosphaerellaceae_genus* | **0.0000** | 0.1069 | 0.1623 | 0.3951 | 0.7128 |
| *unclassified_Capnodiales_family* | **0.0351** | 0.0748 | 0.8141 | 0.2057 | **0.0019** |
| *unclassified_Capnodiales_genus* | **0.0367** | 0.0936 | 0.8141 | 0.3214 | **0.0016** |
| *Dothideales* | 0.0583 | **0.0348** | 0.7476 | 0.5865 | 0.9618 |
| *Dothioraceae* | **0.0250** | **0.0354** | 0.9635 | 0.4691 | 0.7191 |
| *unclassified_Dothioraceae_genus* | 0.0561 | 0.0589 | 0.9419 | 0.3625 | 0.3099 |
| *Dothideomycetes_order_incertae_sedis* | **0.0446** | 0.9517 | 0.2221 | 0.7672 | 0.5571 |
| *Dothideomycetes_family_incertae_sedis* | **0.0430** | 0.9251 | 0.2946 | 0.7615 | 0.6040 |
| *Epicoccum* | **0.0452** | 0.9283 | 0.2946 | 0.7490 | 0.5575 |
| *Pleosporales* | **0.0000** | 0.9630 | 0.2679 | 0.8058 | 0.5338 |
| *Montagnulaceae* | **0.0037** | 0.7200 | 0.7616 | 0.7741 | 0.2930 |
| *unclassified_Montagnulaceae_genus* | **0.0075** | 0.7516 | 0.6781 | 0.6744 | 0.1785 |
| *Phaeosphaeriaceae* | **0.0174** | 0.4555 | 0.8601 | 0.7779 | **0.0006** |
| *Pleosporaceae* | **0.0000** | 0.9220 | 0.2285 | 0.7720 | **0.0369** |
| *Alternaria* | **0.0000** | 0.9422 | 0.1271 | 0.7149 | 0.0510 |
| *Cochliobolus* | **0.0001** | 0.7721 | 0.5482 | 0.4740 | **0.0000** |
| *Exserohilum* | **0.0014** | 0.6062 | 0.7101 | 0.7086 | **0.0001** |
| *Lewia* | 0.1398 | 0.7730 | 0.9998 | 0.7996 | 0.6724 |
| *Stagonospora* | **0.0038** | 0.3333 | 0.3116 | 0.9419 | 0.9369 |
| *Pleosporales_family_incertae_sedis* | **0.0000** | 0.4950 | 0.1313 | 0.7538 | 0.7531 |
| *Ascochyta* | **0.0008** | 0.9336 | 0.0587 | 0.9941 | 0.1543 |
| *Leptosphaerulina* | 0.4286 | 0.0666 | 0.9699 | 0.5129 | 0.3035 |
| *Phoma* | **0.0000** | 0.7698 | 0.1896 | 0.6975 | 0.0968 |
| *unclassified_Pleosporales_family* | **0.0000** | 0.9077 | 0.1283 | 0.2525 | 0.5199 |
| **Phylum** | **Healthy (Dog)** | **Healthy (Body Site)** | **Allergic (Dog)** | **Allergic (Body Site)** | **Shared Sites (Health Status)** |
| *unclassified_Pleosporales_genus* | **0.0000** | 0.9145 | 0.1283 | 0.3156 | 0.4773 |
| *unclassified_Dothideomycetes_order* | **0.0002** | 0.2754 | 0.2231 | 0.7278 | **0.0006** |
| *unclassified_Dothideomycetes_family* | **0.0002** | 0.2504 | 0.2656 | 0.7464 | **0.0005** |
| *unclassified_Dothideomycetes_genus* | **0.0002** | 0.2459 | 0.2324 | 0.7135 | **0.0005** |
| *Eurotiomycetes* | **0.0000** | 0.5979 | 0.2456 | 0.1213 | **0.0092** |
| *Chaetothyriales* | **0.0041** | 0.6878 | 0.7999 | 0.5146 | **0.0005** |
| *Herpotrichiellaceae* | **0.0048** | 0.8447 | 0.9560 | 0.4630 | **0.0014** |
| *unclassified_Chaetothyriales_family* | **0.0057** | 0.1468 | 0.8742 | 0.7677 | 0.0541 |
| *unclassified_Chaetothyriales_genus* | **0.0059** | 0.1501 | 0.9518 | 0.7852 | 0.0516 |
| *Eurotiales* | **0.0000** | 0.9337 | 0.2410 | 0.0928 | **0.0367** |
| *Trichocomaceae* | **0.0000** | 0.9259 | 0.2812 | 0.1160 | **0.0349** |
| *Aspergillus* | **0.0002** | 0.9243 | 0.2885 | 0.5112 | 0.2612 |
| *Penicillium* | **0.0014** | 0.7607 | 0.9969 | 0.3440 | **0.0015** |
| *unclassified_Trichocomaceae_genus* | **0.0003** | 0.9327 | 0.8712 | 0.5862 | **0.0001** |
| *Lecanoromycetes* | **0.0017** | 0.2251 | 0.8360 | 0.4780 | **0.0244** |
| *Leotiomycetes* | **0.0358** | 0.4503 | 0.1580 | 0.3347 | **0.0006** |
| *Helotiales* | **0.0251** | 0.1977 | 0.2731 | 0.5594 | 0.4233 |
| *Helotiaceae* | 0.3777 | 0.1135 | 0.4003 | 0.6162 | 0.7460 |
| *Articulospora* | **0.0280** | 0.1036 | 0.3333 | 0.7147 | 0.2223 |
| *Sclerotiniaceae* | **0.0046** | 0.6983 | 0.6894 | 0.7478 | **0.0002** |
| *unclassified_Sclerotiniaceae_genus* | **0.0042** | 0.6609 | 0.6744 | 0.6816 | **0.0001** |
| *Leotiomycetes_order_incertae_sedis* | **0.0147** | 0.4885 | 0.6958 | 0.8131 | **0.0000** |
| *Erysiphaceae* | **0.0185** | 0.5353 | 0.7018 | 0.7601 | **0.0000** |
| *Blumeria* | **0.0021** | 0.8089 | 0.7194 | 0.7962 | **0.0000** |
| *Golovinomyces* | **0.0098** | **0.0475** | 0.6863 | 0.5598 | **0.0000** |
| *Podosphaera* | **0.0010** | 0.7618 | 0.9999 | 0.7789 | 0.0890 |
| *Pezizomycetes* | **0.0007** | 0.2667 | 0.6075 | 0.6606 | **0.0005** |
| *Pezizales* | **0.0005** | 0.2627 | 0.5895 | 0.7260 | **0.0004** |
| *unclassified_Pezizales_family* | **0.0001** | 0.4901 | 0.8877 | 0.7711 | **0.0002** |
| *unclassified_Pezizales_genus* | **0.0001** | 0.4648 | 0.9641 | 0.6940 | **0.0002** |
| *Saccharomycetes* | **0.0146** | 0.5894 | 0.0801 | 0.5224 | 0.1478 |
| *Saccharomycetales* | **0.0135** | 0.6621 | 0.1803 | 0.5567 | 0.1108 |
| *Saccharomycetaceae* | **0.0357** | 0.9158 | 0.3604 | 0.9771 | **0.0124** |
| *Saccharomycetales_family_incerta_sedis* | **0.0237** | 0.4405 | 0.1507 | 0.5682 | **0.0020** |
| *Candida* | **0.0173** | 0.5950 | 0.1451 | 0.6395 | **0.0014** |
| *Sordariomycetes* | 0.2881 | 0.3269 | 0.2731 | 0.1351 | 0.5468 |
| *Glomerellales* | **0.0088** | **0.0188** | 0.9538 | 0.5077 | **0.0005** |
| *Glomerellales_family_incertae_sedis* | **0.0002** | 0.4989 | 0.8363 | 0.7143 | 0.2873 |
| *Plectosphaerellaceae* | **0.0046** | 0.0801 | 0.9423 | 0.4680 | **0.0004** |
| *Plectosphaerella* | **0.0049** | 0.0916 | 0.9405 | 0.3716 | **0.0010** |
| *Hypocreales* | **0.0087** | 0.2736 | 0.6133 | 0.0767 | 0.2805 |
| *Bionectriaceae* | **0.0055** | 0.4392 | 0.8686 | 0.5152 | 0.1149 |
| **Phylum** | **Healthy (Dog)** | **Healthy (Body Site)** | **Allergic (Dog)** | **Allergic (Body Site)** | **Shared Sites (Health Status)** |
| *Hydropisphaera* | 0.1597 | 0.3533 | 0.5457 | 0.4012 | **0.0033** |
| *Clavicipitaceae* | **0.0001** | 0.6948 | 0.7772 | 0.1347 | **0.0002** |
| *Claviceps* | **0.0000** | 0.7728 | 0.6874 | 0.2892 | **0.0002** |
| *Hypocreales_family_incertae_sedis* | **0.0021** | 0.0962 | 0.6056 | 0.2733 | 0.8023 |
| *Fusarium* | **0.0010** | 0.1086 | 0.7275 | 0.3775 | 0.3147 |
| *Myrothecium* | **0.0164** | 0.1485 | 0.9756 | 0.3224 | 0.2075 |
| *Sarocladium* | **0.0166** | 0.2437 | 0.1436 | 0.6888 | 0.4185 |
| *unclassified_Hypocreales_family* | **0.0002** | 0.2341 | 0.4727 | 0.1742 | **0.0368** |
| *unclassified_Hypocreales_genus* | **0.0002** | 0.2341 | 0.5252 | 0.2177 | **0.0335** |
| *Microascales* | **0.0000** | 0.2532 | 0.2405 | 0.5063 | **0.0001** |
| *Halosphaeriaceae* | **0.0000** | 0.7699 | 0.6194 | 0.5469 | **0.0000** |
| *Periconia* | **0.0000** | 0.7812 | 0.6750 | 0.4625 | **0.0000** |
| *Sordariales* | 0.0858 | 0.3011 | 0.4976 | 0.6379 | 0.1058 |
| *Chaetomiaceae* | 0.2769 | 0.0766 | 0.8717 | 0.4957 | 0.2901 |
| *Chaetomium* | 0.5055 | 0.0566 | 0.9395 | 0.3636 | 0.3267 |
| *Sordariaceae* | **0.0214** | 0.7725 | 0.2388 | 0.5482 | **0.0120** |
| *Gelasinospora* | **0.0134** | 0.8033 | 0.3318 | 0.3825 | **0.0081** |
| *Sordariomycetes_order_incertae_sedis* | **0.0000** | 0.9384 | 0.1973 | 0.7177 | **0.0017** |
| *Magnaporthaceae* | **0.0000** | 0.7571 | 0.1421 | 0.7803 | 0.6100 |
| *Magnaporthe* | **0.0000** | 0.7337 | 0.1171 | 0.9354 | 0.9896 |
| *Phialophora* | **0.0004** | 0.7299 | 0.7168 | 0.5080 | 0.1649 |
| *Sordariomycetes_family_incertae_sedis* | **0.0093** | 0.6970 | 0.4004 | 0.3548 | **0.0002** |
| *Acremonium* | **0.0092** | 0.7437 | 0.4004 | 0.3411 | **0.0001** |
| *Trichosphaeriales* | 0.9909 | 0.1596 | 0.7492 | 0.6001 | 0.3456 |
| *unclassified_Sordariomycetes_order* | 0.1164 | 0.2628 | 0.7664 | 0.4912 | **0.0261** |
| *unclassified_Sordariomycetes_family* | 0.1231 | 0.3212 | 0.8211 | 0.5668 | **0.0235** |
| *unclassified_Sordariomycetes_genus* | 0.1231 | 0.3170 | 0.7983 | 0.3543 | **0.0202** |
| *Xylariales* | **0.0005** | 0.4756 | 0.6538 | 0.5695 | 0.2086 |
| *Amphisphaeriaceae* | 0.5628 | 0.2641 | 0.4741 | 0.4549 | 0.2571 |
| *Pestalotiopsis* | 0.6466 | 0.3145 | 0.6994 | 0.3849 | 0.2654 |
| *unclassified_Xylariales_family* | 0.2854 | 0.5063 | 0.8605 | 0.4542 | **0.0010** |
| *unclassified_Xylariales_genus* | 0.2803 | 0.5063 | 0.9322 | 0.3650 | **0.0009** |
| *Xylariaceae* | **0.0000** | 0.6987 | 0.5380 | 0.7173 | 0.7921 |
| *unclassified_Ascomycota_class* | 0.0924 | 0.2859 | 0.8409 | 0.0596 | 0.4205 |
| *unclassified_Ascomycota_order* | 0.0910 | 0.2859 | 0.7568 | 0.1341 | 0.4055 |
| *unclassified_Ascomycota_family* | 0.0951 | 0.3064 | 0.8226 | 0.2235 | 0.4113 |
| *unclassified_Ascomycota_genus* | 0.0963 | 0.2979 | 0.8446 | 0.2794 | 0.3877 |
| *Basidiomycota* | **0.0009** | 0.8417 | 0.2303 | 0.3913 | 1.1801 |
| *Agaricomycetes* | **0.0008** | 0.6656 | 0.1815 | 0.3777 | 0.9390 |
| *Agaricales* | **0.0006** | 0.6864 | 0.9271 | 0.5457 | 0.1355 |
| *Agaricales_family_incertae_sedis* | **0.0000** | 0.9116 | 0.6315 | 0.7417 | 0.1400 |
| *Psathyrellaceae* | **0.0019** | 0.7344 | 0.5334 | 0.5467 | 0.2371 |
| **Phylum** | **Healthy (Dog)** | **Healthy (Body Site)** | **Allergic (Dog)** | **Allergic (Body Site)** | **Shared Sites (Health Status)** |
| *Coprinellus* | 0.0712 | 0.7759 | 0.6878 | 0.3865 | 0.7973 |
| *unclassified_Psathyrellaceae_genus* | **0.0007** | 0.7713 | 0.5526 | 0.7153 | 0.9682 |
| *Schizophyllaceae* | 0.4748 | 0.9379 | 0.6039 | 0.9790 | **0.0002** |
| *Schizophyllum* | 0.2798 | 0.9368 | 0.6672 | 0.9797 | **0.0002** |
| *unclassified_Agaricales_family* | 0.0578 | 0.1806 | 0.6301 | 0.7298 | **0.0155** |
| *unclassified_Agaricales_genus* | 0.0592 | 0.1736 | 0.6632 | 0.7057 | **0.0127** |
| *Agaricomycetes_order_incertae_sedis* | **0.0003** | 0.6643 | 0.4680 | 0.9197 | 0.8748 |
| *Corticiaceae* | **0.0004** | 0.8797 | 0.9479 | 0.7402 | 0.6067 |
| *Peniophoraceae* | **0.0041** | 0.7819 | 0.8786 | 0.7335 | 0.8150 |
| *Peniophora* | **0.0057** | 0.8152 | 0.7116 | 0.7399 | 0.9402 |
| *Auriculariales* | **0.0000** | 0.9995 | 0.5531 | 0.5858 | 0.1068 |
| *Cantharellales* | **0.0075** | 0.6450 | 0.7711 | 0.8314 | **0.0012** |
| *Ceratobasidiaceae* | 0.4079 | 0.6024 | 0.5924 | 0.7751 | 0.7808 |
| *Hydnaceae* | **0.0000** | 0.9972 | 0.3466 | 0.7771 | **0.0000** |
| *Hydnum* | **0.0000** | 0.9972 | 0.3507 | 0.7369 | **0.0000** |
| *Polyporales* | **0.0005** | 0.7433 | 0.7418 | 0.7187 | 0.4230 |
| *Polyporales_family_incertae_sedis* | **0.0032** | 0.7164 | 0.8215 | 0.7881 | 0.2861 |
| *Irpex* | **0.0000** | 0.8087 | 0.7062 | 0.9227 | **0.0031** |
| *Trametes* | 0.0979 | 0.5037 | 0.6774 | 0.3449 | **0.0305** |
| *Basidiomycota_class_incertae_sedis* | **0.0008** | 0.6310 | 0.7988 | 0.4996 | 0.2759 |
| *Basidiomycota_order_incertae_sedis* | **0.0007** | 0.7027 | 0.3742 | 0.7414 | **0.0003** |
| *Basidiomycota_family_incertae_sedis* | **0.0009** | 0.7353 | 0.4252 | 0.7479 | **0.0002** |
| *Cerinosterus* | **0.0002** | 0.8613 | 0.7108 | 0.5969 | **0.0000** |
| *Wallemia* | **0.0000** | 0.8556 | 0.3587 | 0.7954 | **0.0022** |
| *Entylomatales* | **0.0000** | 0.9982 | 0.4933 | 0.9688 | **0.0021** |
| *unclassified_Entylomatales_family* | **0.0000** | 0.9508 | 0.5851 | 0.9733 | **0.0019** |
| *unclassified_Entylomatales_genus* | **0.0000** | 0.9476 | 0.6206 | 0.9864 | **0.0016** |
| *Malasseziales* | **0.0491** | 0.7898 | 0.5119 | 0.9775 | 0.3096 |
| *Malasseziales_family_incertae_sedis* | **0.0484** | 0.7953 | 0.6003 | 0.9891 | 0.3000 |
| *Malassezia* | 0.0502 | 0.7697 | 0.6331 | 0.9752 | 0.3043 |
| *Pucciniales* | 0.1532 | 0.6740 | 0.2342 | 0.7959 | **0.0000** |
| *Pucciniastraceae* | **0.0343** | 0.7176 | 0.1882 | 0.7643 | **0.0000** |
| *Pucciniastrum* | **0.0360** | 0.7662 | 0.1177 | 0.7287 | **0.0000** |
| *Sporidiobolales* | **0.0043** | 0.4381 | 0.7232 | 0.4927 | 0.0691 |
| *Sporidiobolales_family_incertae_sedis* | **0.0027** | 0.4608 | 0.8307 | 0.5192 | **0.0366** |
| *Rhodotorula* | **0.0485** | 0.3564 | 0.7740 | 0.4093 | 0.1312 |
| *Sporobolomyces* | **0.0002** | 0.7595 | 0.3268 | 0.4636 | **0.0001** |
| *unclassified_Sporidiobolales_family* | 0.2733 | 0.4264 | 0.9414 | 0.5908 | **0.0196** |
| *unclassified_Sporidiobolales_genus* | 0.2662 | 0.3553 | 0.9779 | 0.5043 | **0.0163** |
| *Tremellomycetes* | **0.0079** | 0.6093 | 0.3217 | 0.3105 | 0.1561 |
| *Filobasidiales* | 0.9636 | 0.3700 | 0.2608 | 0.5012 | 0.2034 |
| *Filobasidiaceae* | 0.9321 | 0.6890 | 0.3477 | 0.4594 | 0.2365 |
| **Phylum** | **Healthy (Dog)** | **Healthy (Body Site)** | **Allergic (Dog)** | **Allergic (Body Site)** | **Shared Sites (Health Status)** |
| *Filobasidium* | 0.9321 | 0.7509 | 0.3431 | 0.3961 | 0.2094 |
| *Tremellales* | **0.0005** | 0.7956 | 0.2591 | 0.4853 | 0.1108 |
| *Tremellaceae* | **0.0037** | 0.5953 | 0.4053 | 0.7157 | 0.1233 |
| *Tremellales_family_incertae_sedis* | **0.0056** | 0.6909 | 0.3334 | 0.4441 | 0.1037 |
| *Cryptococcus* | **0.0042** | 0.7766 | 0.3561 | 0.4140 | 0.0837 |
| *unclassified_Tremellales_family* | **0.0002** | 0.4065 | 0.8451 | 0.5855 | 0.7601 |
| *unclassified_Tremellales_genus* | **0.0002** | 0.3455 | 0.8594 | 0.5053 | 0.7436 |
| *unclassified_Basidiomycota_class* | **0.0046** | 0.4873 | 0.2681 | 0.9499 | 0.6863 |
| *unclassified_Basidiomycota_order* | **0.0043** | 0.4797 | 0.2585 | 0.9770 | 0.6551 |
| *unclassified_Basidiomycota_family* | **0.0044** | 0.4920 | 0.3351 | 0.9826 | 0.7065 |
| *unclassified_Basidiomycota_genus* | **0.0043** | 0.5158 | 0.3427 | 0.9895 | 0.6723 |
| *Ustilaginomycetes* | **0.0000** | 0.6175 | 0.8904 | 0.1708 | 0.3354 |
| *Ustilaginales* | **0.0000** | 0.6385 | 0.8217 | 0.2609 | 0.3279 |
| *unclassified_Ustilaginales_family* | **0.0000** | 0.7361 | 0.9735 | 0.3847 | 0.3609 |
| *unclassified_Ustilaginales_genus* | **0.0000** | 0.7729 | 0.9876 | 0.3306 | 0.3308 |
| *Ustilaginaceae* | **0.0000** | 0.7854 | 0.7909 | 0.3119 | 0.2873 |
| *Pseudozyma* | **0.0001** | 0.7941 | 0.8725 | 0.4301 | 0.3832 |
| *Sporisorium* | **0.0000** | 0.5984 | 0.4710 | 0.3324 | **0.0000** |
| *unclassified_Ustilaginaceae_genus* | **0.0031** | 0.6630 | 0.4016 | 0.7883 | 0.3122 |
| *Glomeromycota* | 0.1166 | 1.2023 | 0.8817 | 0.5524 | 0.4158 |
| *Glomeromycetes* | 0.1244 | 0.4809 | 0.8817 | 0.4420 | 0.2661 |
| *unclassified_Fungi_phylum* | **0.0003** | 0.4225 | 0.2804 | 0.6427 | **0.0412** |
| *unclassified_Fungi_class* | **0.0003** | 0.6760 | 0.2243 | 0.5142 | **0.0264** |
| *unclassified_Fungi_order* | **0.0002** | 0.6613 | 0.2524 | 0.5784 | **0.0247** |
| *unclassified_Fungi_family* | **0.0002** | 0.6852 | 0.3059 | 0.5713 | **0.0215** |
| *unclassified_Fungi_genus* | **0.0002** | 0.7369 | 0.3004 | 0.5211 | **0.0182** |
| Total number of significant taxa (p<0.05) | 153 | 4 | **0** | **0** | 85 |

Supplementary Table S6. Alpha diversity calculations by site in allergic skin group.

| **Skin Site** | **Observed Species** | | **Chao1** | | **Shannon** | | **Inverse Simpson** | |
| --- | --- | --- | --- | --- | --- | --- | --- | --- |
|  | Median | Min-Max | Median | Min-Max | Median | Min-Max | Median | Min-Max |
| Axilla | 34 | 20-52 | 48 | 42-69 | 1.8 | 1.4-2.3 | 3.6 | 2.7-6.2 |
| Ear | 21 | 12-31 | 39 | 22-59 | 1.4 | 0.1-1.9 | 2.6 | 1.0-5.4 |
| Groin | 27 | 23-47 | 48 | 43-56 | 1.9 | 0.3-2.5 | 3.7 | 1.1-7.5 |
| Interdigital | 38 | 31-45 | 52 | 46-59 | 2.1 | 1.0-5.6 | 4.7 | 1.8-9.1 |
| Lumbar | 27 | 21-37 | 47 | 31-63 | 2.0 | 1.2-2.4 | 4.8 | 2.5-7.4 |
| Nostril | 19 | 16-21 | 38 | 29-42 | 1.2 | 0.2-1.9 | 2.8 | 1.1-5.8 |

Supplementary Table S7. Alpha diversity calculations by dog in allergic skin group.

| **Dog Number** | **Observed Species** | | **Chao1** | | **Shannon** | | **Inverse Simpson** | |
| --- | --- | --- | --- | --- | --- | --- | --- | --- |
|  | Median | Min-Max | Median | Min-Max | Median | Min-Max | Median | Min-Max |
| 11 | 31 | 16-52 | 43 | 29-69 | 2.0 | 1.5-2.5 | 4.7 | 3.2-7.7 |
| 12 | 31 | 20-43 | 42 | 37-54 | 2.1 | 1.4-2.3 | 5.1 | 2.6-6.2 |
| 13 | 23 | 20-37 | 46 | 39-51 | 1.6 | 0.3-2.6 | 3.8 | 1.1-9.1 |
| 14 | 26 | 21-45 | 49 | 38-59 | 1.6 | 0.4-2.6 | 3.1 | 1.1-7.5 |
| 15 | 22 | 20-39 | 40 | 31-52 | 1.9 | 1.4-2.1 | 4.9 | 2.8-7.1 |
| 16 | 31 | 16-40 | 47 | 32-56 | 1.4 | 0.7-1.5 | 2.6 | 1.5-3.1 |
| 17 | 37 | 12-39 | 48 | 22-51 | 1.6 | 0.1-1.9 | 3.0 | 1.0-3.7 |
| 18 | 24 | 19-38 | 46 | 38-63 | 1.8 | 1.2-2.2 | 4.2 | 2.8-4.8 |

Supplementary Table S8. Dissimilarity of fungal community membership and structure between body sites.

|  |  | | | **Bray Curtis** | | **Jaccard** | **Theta YC** | |  |
| --- | --- | --- | --- | --- | --- | --- | --- | --- | --- |
| Allergic Dogs | | Nostril | Axilla | | 0.331* | 0.207 | | 0.294* | |
|  |  |  | Groin | | 0.227 | 0.151 | | 0.213* | |
|  |  |  | Interdigital | | 0.441* | 0.176 | | 0.300* | |
| Comparison of Shared Sites between Healthy and Allergic Dogs | | Ear | | | 0.093 | 0.249* | | 0.092 | |
|  |  | Groin | | | 0.013 | 0.264* | | 0.052 | |
|  |  | Interdigital | | | -0.022 | 0.402* | | -0.033 | |
