# Supplementary figures and images for: What is living on your dog's skin? Characterization of the canine cutaneous mycobiota and fungal dysbiosis in canine allergic dermatitis
Source: FEMS Microbiol Ecol. 2015 Nov 5;91(12):fiv139. doi: 10.1093/femsec/fiv139 (PMC4657189; doi:10.1093/femsec/fiv139)

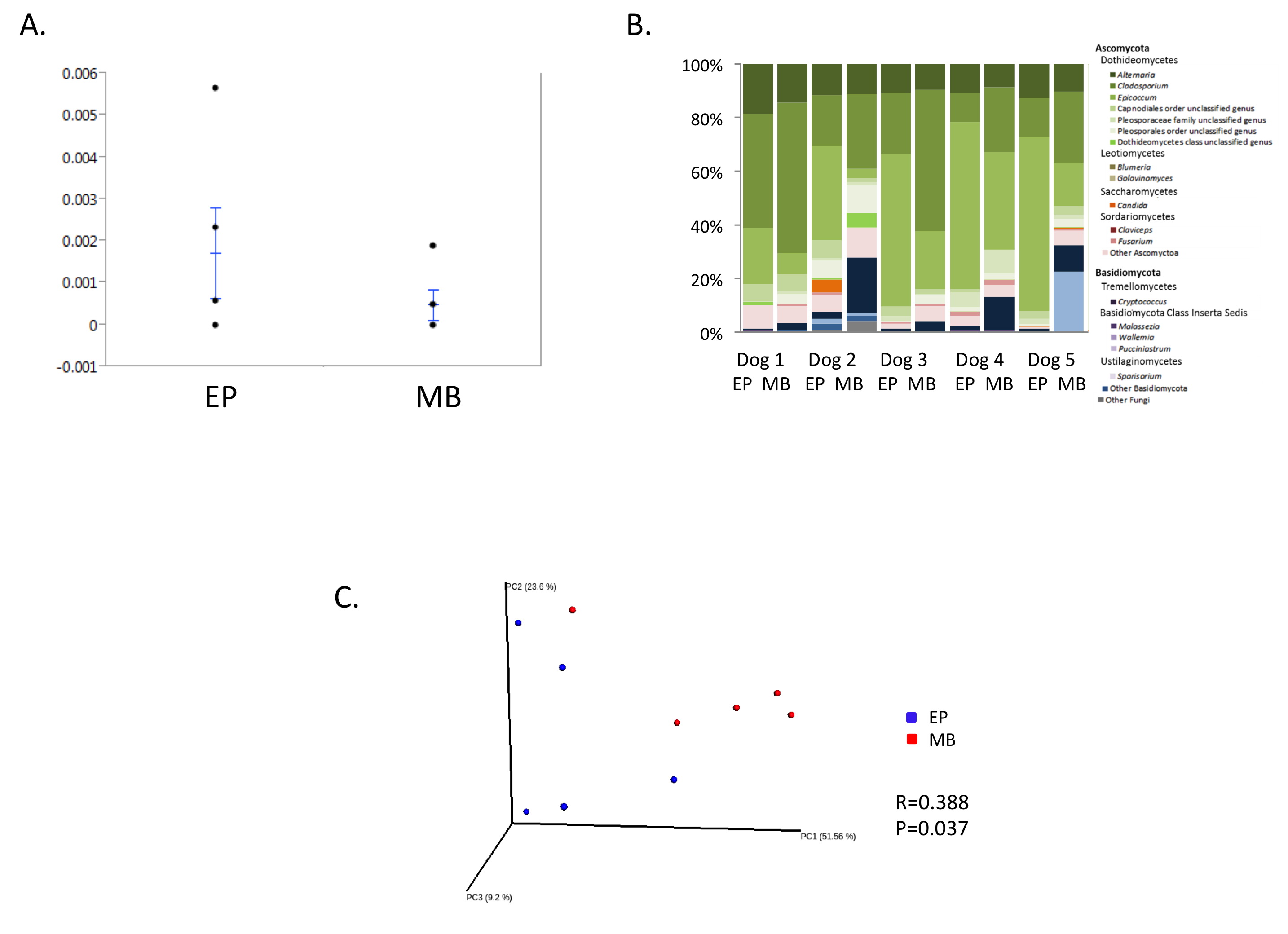

Supplement: Supplementary data are available at FEMSEC online [file Supplement_Materials.zip › Figure S1.jpg]

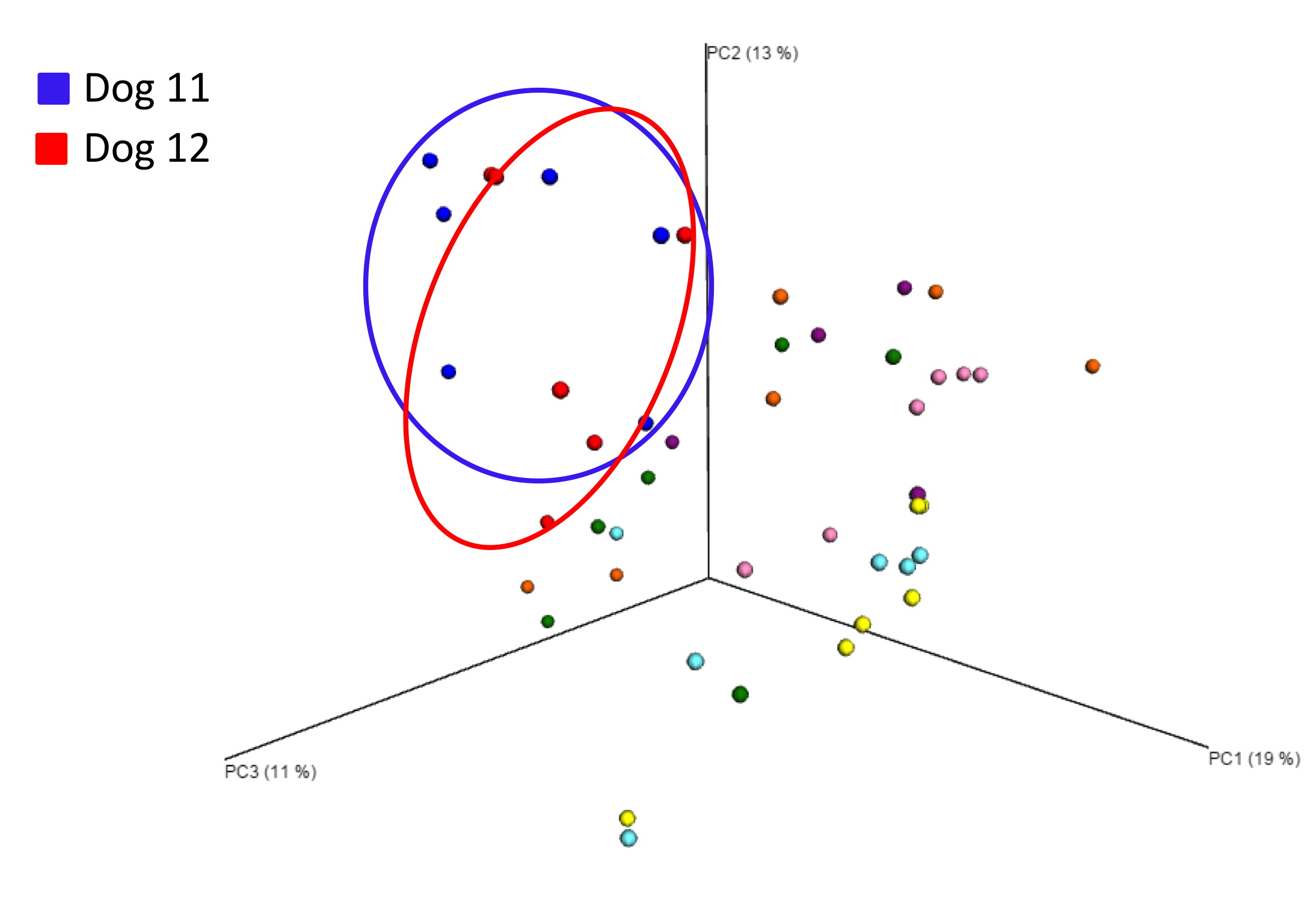

Supplement: Supplementary data are available at FEMSEC online [file Supplement_Materials.zip › Figure S2.jpg]

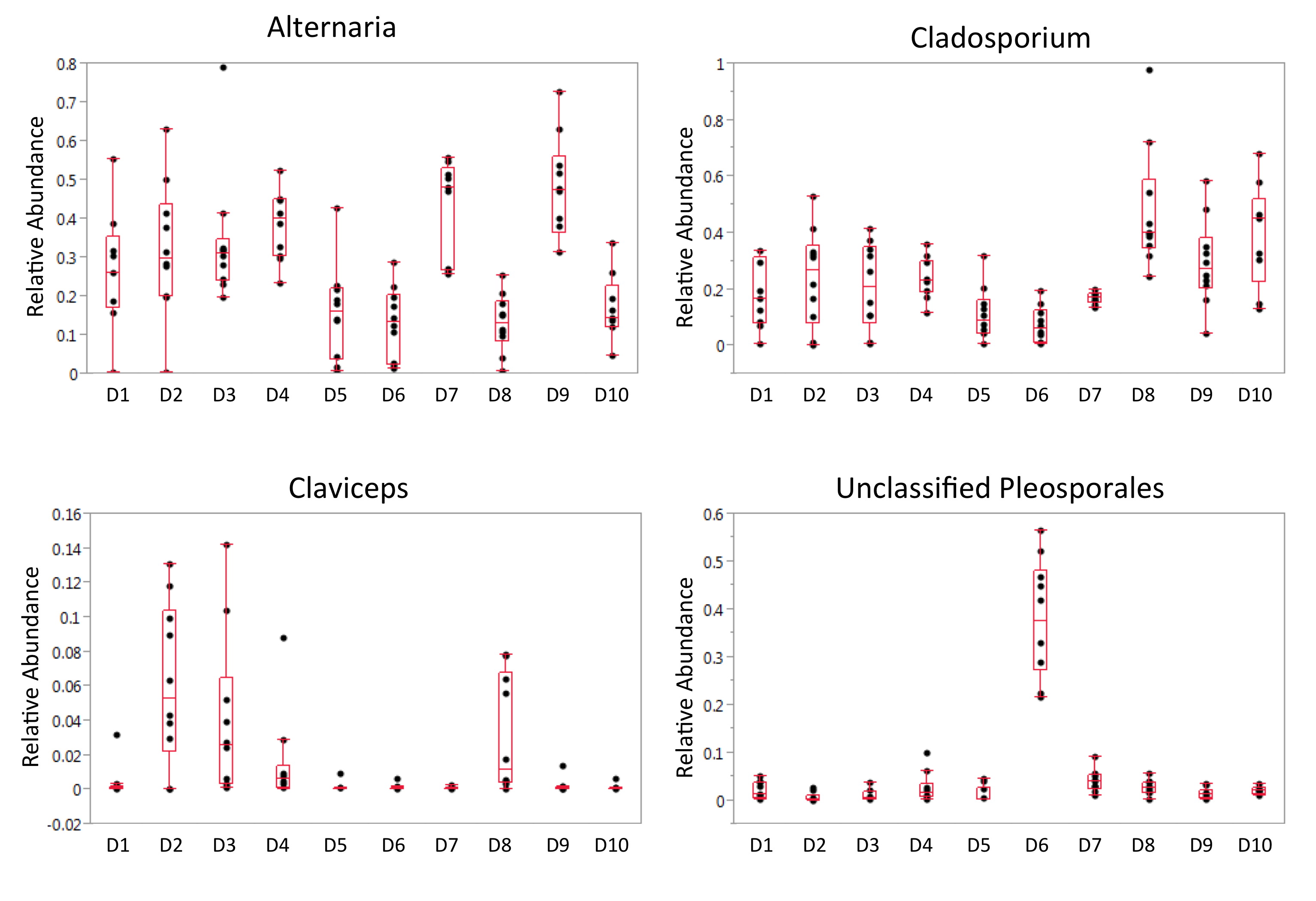

Supplement: Supplementary data are available at FEMSEC online [file Supplement_Materials.zip › Figure S3.jpg]
