# Supplementary material for: What is living on your dog's skin? Characterization of the canine cutaneous mycobiota and fungal dysbiosis in canine allergic dermatitis
Source: FEMS Microbiol Ecol. 2015 Nov 5;91(12):fiv139. doi: 10.1093/femsec/fiv139 (PMC4657189; doi:10.1093/femsec/fiv139)
Supplement: Supplementary data are available at FEMSEC online [file Supplement_Materials.zip › Legends.doc]

Supplementary Table S1. Overall effect of body site, dog, and health status on alpha diversity. Fungal richness was estimated with observed species and Chao1 in Mothur, fungal diversity was estimated using the non-parametric Shannon and Inverse Simpson in Mothur, and overall effect of body site, dog and health status was calculated with Kruskal-Wallis tests performed in JMP. Sample statistics and P-values are listed for each factor on each group of samples including healthy only body sites, allergic only body sites, and body sites shared between health status groups.

Supplementary Table S2. Alpha diversity calculations by site in healthy skin group. Fungal richness was estimated with observed species and Chao1 in Mothur, and fungal diversity was estimated using the non-parametric Shannon and Inverse Simpson in Mothur. The median, minimum and maximum values were calculated for each healthy body site.

Supplementary Table S3. Alpha diversity calculations by dog in healthy skin group. Fungal richness was estimated with observed species and Chao1 in Mothur, and fungal diversity was estimated using the non-parametric Shannon and Inverse Simpson in Mothur. The median, minimum and maximum values were calculated for each healthy dog.

Supplementary Table S4. Dissimilarity of fungal community membership and structure between dogs. The median R-value was calculated from R vales only for comparisons that were significant. The minimum and maximum R-values for this subset of significant R-values were also calculated and reported within the table. ANOSIM was performed on distance matrices produced using the three beta diversity metrics on two groups of samples: healthy only and allergic only. If there were no significant comparisons then n/a was listed in the table. All P-values were corrected using the FDR equation.

Supplementary Table S5. Combined and filtered relative abundance table for 193 fungal taxa that were tested for significant differences between body sites, dogs, and health status. For each test, the Kruskal-Wallis P-values are listed in columns following the taxa names. The relative abundance of each of these taxa are also listed for each sample (n=144). Classification of each sample within body site, dog, and health status are found in rows above the sample identifiers.

Supplementary Table S6. Alpha diversity calculations by site in allergic skin group. Fungal richness was estimated with Observed species and Chao1 in Mothur, and fungal diversity was estimated using the non-parametric Shannon and Inverse Simpson in Mothur. The median, minimum and maximum values were calculated for each allergic body site.

Supplementary Table S7. Alpha diversity calculations by dog in allergic skin group. Fungal richness was estimated with Observed species and Chao1 in Mothur, and fungal diversity was estimated using the non-parametric Shannon and Inverse Simpson in Mothur. The median, minimum and maximum values were calculated for each allergic dog.

Supplementary Table S8. Dissimilarity of fungal community membership and structure between body sites. R values from global test of the factor body site performed with ANOSIM on distance matrices generated from only allergic body sites using the three beta diversity metrics Bray Curtis, Jaccard, and Yue-Clayton theta coefficient (Mothur) were listed for significant comparisons (*P<0.05 after FDR correction). R values from global test of the factor health status performed with ANOSIM on distance matrices generated from only body sites shared between the two health status groups using the three beta diversity metrics Bray Curtis, Jaccard, and Yue-Clayton theta coefficient (Mothur) were listed for significant comparisons (*P<0.05 after FDR correction).

Supplementary Figure S1 Results from comparison of two extraction protocols. (a) Relative abundances of *Malassezia* in the ear canal of five dogs were plotted in JMP with mean error bars. EP (Epicentre MasterPure Yeast Lysis Kit) signifies the samples extracted using the Findley *et. al.* 2013 protocol, and MB (MoBio) signifies the samples extracted following the Rodrigues *et. al.* 2014 protocol. (b) Stacked bar plot of predominant fungal taxa for each sample, grouped by dog number and extraction protocol. S represents the taxa identified for the swab only control. (c) PCoA plot of Bray Curtis calculated pairwise distances between samples. Samples are colored by kit or negative control.

Supplementary Figure S2. Dissimilarity of fungal community membership between allergic dogs. PCoA plot of Bray Curtis calculated pairwise distances between body sites of only allergic dogs. The two dogs in red and blue cluster together and come from the same household. Each color represents the mycobiota from body sites on an individual dog.

Supplementary Figure S3. Relative abundances of fungal taxa that were significantly different between dogs. Dots represent body sites and are grouped by dog.
